# TRK-Fused Gene (TFG), a protein involved in protein secretion pathways, is an essential component of the antiviral innate immune response

**DOI:** 10.1371/journal.ppat.1009111

**Published:** 2021-01-07

**Authors:** Kashif Aziz Khan, Alexandre Marineau, Priscilla Doyon, Mariana Acevedo, Étienne Durette, Anne-Claude Gingras, Marc J. Servant

**Affiliations:** 1 Faculty of Pharmacy, Université de Montréal, Montréal, Canada; 2 Lunenfeld-Tanenbaum Research Institute at Mount Sinai Hospital, Toronto, Ontario, Canada; 3 Department of Molecular Genetics, University of Toronto, Toronto, Ontario, Canada; National Institute of Allergy and Infectious Diseases, UNITED STATES

## Abstract

Antiviral innate immune response to RNA virus infection is supported by Pattern-Recognition Receptors (PRR) including RIG-I-Like Receptors (RLR), which lead to type I interferons (IFNs) and IFN-stimulated genes (ISG) production. Upon sensing of viral RNA, the E3 ubiquitin ligase TNF Receptor-Associated Factor-3 (TRAF3) is recruited along with its substrate TANK-Binding Kinase (TBK1), to MAVS-containing subcellular compartments, including mitochondria, peroxisomes, and the mitochondria-associated endoplasmic reticulum membrane (MAM). However, the regulation of such events remains largely unresolved. Here, we identify TRK-Fused Gene (TFG), a protein involved in the transport of newly synthesized proteins to the endomembrane system via the Coat Protein complex II (COPII) transport vesicles, as a new TRAF3-interacting protein allowing the efficient recruitment of TRAF3 to MAVS and TBK1 following Sendai virus (SeV) infection. Using siRNA and shRNA approaches, we show that TFG is required for virus-induced TBK1 activation resulting in C-terminal IRF3 phosphorylation and dimerization. We further show that the ability of the TRAF3-TFG complex to engage mTOR following SeV infection allows TBK1 to phosphorylate mTOR on serine 2159, a post-translational modification shown to promote mTORC1 signaling. We demonstrate that the activation of mTORC1 signaling during SeV infection plays a positive role in the expression of Viperin, IRF7 and IFN-induced proteins with tetratricopeptide repeats (IFITs) proteins, and that depleting TFG resulted in a compromised antiviral state. Our study, therefore, identifies TFG as an essential component of the RLR-dependent type I IFN antiviral response.

## Author summary

Antiviral innate immune response is the first line of defence against the invading viruses through type I interferon (IFN) signaling. However, viruses have devised ways to target signaling molecules for aberrant IFN response and worsen the disease outcome. As such, deciphering the roles of new regulators of innate immunity could transform the antiviral treatment paradigm by introducing novel panviral therapeutics designed to reinforce antiviral host responses. This could be of great use in fighting recent outbreaks of severe acute respiratory syndrome coronavirus (SARS-CoV), Middle East respiratory syndrome MERS-CoV, and the more recent SARS-CoV-2 causing the COVID-19 pandemic. However, aberrant activation of such pathways can lead to detrimental consequences, including autoimmune diseases. Regulation of type I IFN responses is thus of paramount importance. To prevent an uncontrolled response, signaling events happen in discrete subcellular compartments, therefore, distinguishing sites involved in recognition of pathogens and those permitting downstream signaling. Here, we show TFG as a new regulator of type I IFN response allowing the efficient organization of signaling molecules. TFG, thus, further substantiates the importance of the protein trafficking machinery in the regulation of optimal antiviral responses. Our findings have implications for both antiviral immunity and autoimmune diseases.

## Introduction

The cellular antiviral innate immune response against invading pathogens represents a critical step in maintaining cell homeostasis and host survival. Thus, understanding molecular determinants governing the optimal organization of the antiviral innate immune response remains essential to identify novel cellular targets for future antiviral or autoimmune therapies. Generally, the establishment of such potent antiviral response relies on the detection of viral nucleic acid by evolutionarily conserved pattern-recognition receptors (PRRs) [1,2]. The recognition of viral components by PRRs triggers multiple pathways that culminate in the activation of multiple latent transcription factors, including interferons regulatory factor 3 (IRF3), as well as nuclear factor κB (NF-κB) [3]. These transcription factors directly enable the expression of several immunomodulatory genes, including the type I interferons (IFNs-α and IFN-β) and IFNs-stimulated genes (ISGs) that disrupt viral replication and dissemination, and mobilize adaptive immunity [4]. Interestingly, only a subset of PRRs can lead to the synthesis of type I IFNs and subsequent expression of ISGs. These receptors include the endosome localized Toll-like receptors (TLRs) TLR3, TLR7, TLR8, TLR9, the cytosolic cyclic GMP-AMP synthase (cGAS) as well as retinoic acid-inducible gene-I (RIG-I)-like receptors (RLRs), RIG-I, and melanoma differentiation-associated gene 5 (MDA5) [5–8]. Yet, even if these TLRs are involved in the detection of extracellular viral nucleic acids of key immune cells, most other cell types, such as epithelial cells and fibroblasts, rely mainly on cytosolic RLRs to sense RNA replication intermediates [9–11].

RIG-I and MDA-5 are closely related proteins that belong to the DExD/H Box helicase family and represent the most important cytoplasmic sensors for viral RNA [6]. Specifically, RIG-I is a sensor for 5’-triphosphate-containing short double-stranded (ds)RNA structures from various single-stranded (ss)RNA viruses including *Sendai virus* (SeV), *hepatitis C virus* (HCV), *vesicular stomatitis virus* (VSV) and *influenza virus* [12]. On the other hand, whereas MDA5 shares certain ligands with RIG-I, such as the synthetic polyinosinic:polycytidylic acid (poly(I:C)), it also detects long dsRNA molecules from different families of viruses such as *Picornaviridae*, *Caliciviridae*, *Coronaviridae* [13,14]. Upon binding to viral RNA, RIG-I and MDA-5 are recruited to the adaptor protein known as mitochondrial antiviral signaling (MAVS) through homotypic interactions between their caspase recruitment domains (CARDs) [15]. RIG-I-MAVS interaction leads to the recruitment of different signaling effectors, thus creating a macromolecular signalosome complex that eventually culminates in the activation of IRF3 [15–19]. Notably, tumor necrosis factor receptor (TNFR)-associated factors (TRAF) family member TRAF3 has been identified as a major effector of the MAVS downstream signaling pathways which activates TANK-binding kinase 1 (TBK1) [20–30]. In this context, TRAF3 is believed to induce the transautophosphorylation of TBK1 on Ser172 [31, 32], followed by the phosphorylation of IRF3, its dimerization and nuclear translocation where it rapidly induces the transcription of type I IFN genes and a subset of ISGs [33–36]. In summary, much has been learned about the effectors of RLR pathways. However, much less is known regarding how such effectors are functionally recruited to one another to initiate rapid and efficient signalling following RLR engagement. This could help to understand the way antiviral networks are incorporated into cellular substructures and stimulate new paradigms in the field of innate immunity.

Recent studies support the role of subcellular synapses, consisting of physical contact sites between organelles, in establishing scaffolds for signal transduction in antiviral immunity. The first evidence that cytosolic RLR signaling may propagate from such interrelated organelles came from studies of MAVS, a transmembrane adaptor with diverse subcellular localization including mitochondria, peroxisomes and mitochondrial-associated ER membrane (MAM), an interface between mitochondria and the ER [37–39]. Depending on its subcellular localization, it has been proposed that MAVS could sustain multiple signaling pathways [39]. Moreover, the discovery of new MAVS-interacting type I IFNs mediators, such as STING, suggested a role for the ER-to-Golgi transport system in innate immunity. Indeed, STING was shown to translocate from the ER to the ERGIC/Golgi apparatus to eventually associate with TBK1 [40–44]. Interestingly, STING trafficking relies on ER-derived Coat Protein complex II (COPII) vesicle coat proteins allowing its maximal signaling capacities at the ERGIC [43,45–47]. In addition to these studies, we previously showed that the ER-to-Golgi vesicular transport system serves as an organizing membrane-rich platform allowing the proper positioning of TRAF3 with MAVS onto the mitochondria network following virus infection [48]. Other recent studies also propose a role of ER-to-Golgi trafficking proteins in TRAF3-mediated antiviral signaling events [26] and a recent study demonstrated the activation of TBK1 at the Golgi apparatus upon viral RNA sensing [49]. Nevertheless, how cells allow TRAF3 to be recruited to such functional antiviral subcellular synapses remains largely unresolved. We sought to determine the mechanism underlying the recruitment of the ER-to-Golgi resident TRAF3 to membrane-bound MAVS for the formation of functional signalling complex upon viral infection.

Here, our group identified a new role for an ER-to-Golgi resident protein, TRK-Fused Gene (TFG), as a TRAF3-interacting protein that positively regulates the RLR-dependent type I IFN antiviral response. TFG was first identified as a fusion partner of the nerve growth factor (NGF) receptor (NTRK1) that generates the papillary thyroid TRK-T3 oncogene following chromosomal arrangement [50]. Subsequently, several other oncogenic fusion proteins involving TFG have been reported [51]. While its molecular function is just starting to be unveiled, the current model suggests that TFG functions in intracellular protein trafficking by regulating the integrity of the ER-Golgi interface [52,53]. It has been shown that TFG promotes the organization of ER exit sites and allows clustering of COPII vesicles between the ER and the ERGIC, allowing rapid movement of secretory cargoes as well as promoting outer coat disassembly of COPII carriers at the ER/ERGIC interface [54–56]. Our results detail the functional role of TFG in innate immunity as an ER-to-Golgi resident protein which allows TRAF3 to interact with upstream adapter protein MAVS and downstream kinase TBK1 resulting in activation of TBK1 upon viral infection. Moreover, we demonstrate that TBK1 also associates and phosphorylates mTOR on serine (Ser) 2159 upon RLR engagement in a TFG-dependent manner. Our study, therefore, identifies TFG as an essential component of type I IFN antiviral response.

## Results

### TFG interacts specifically with TRAF3 and localizes on the ER-to-Golgi compartments

**Fig 1:**
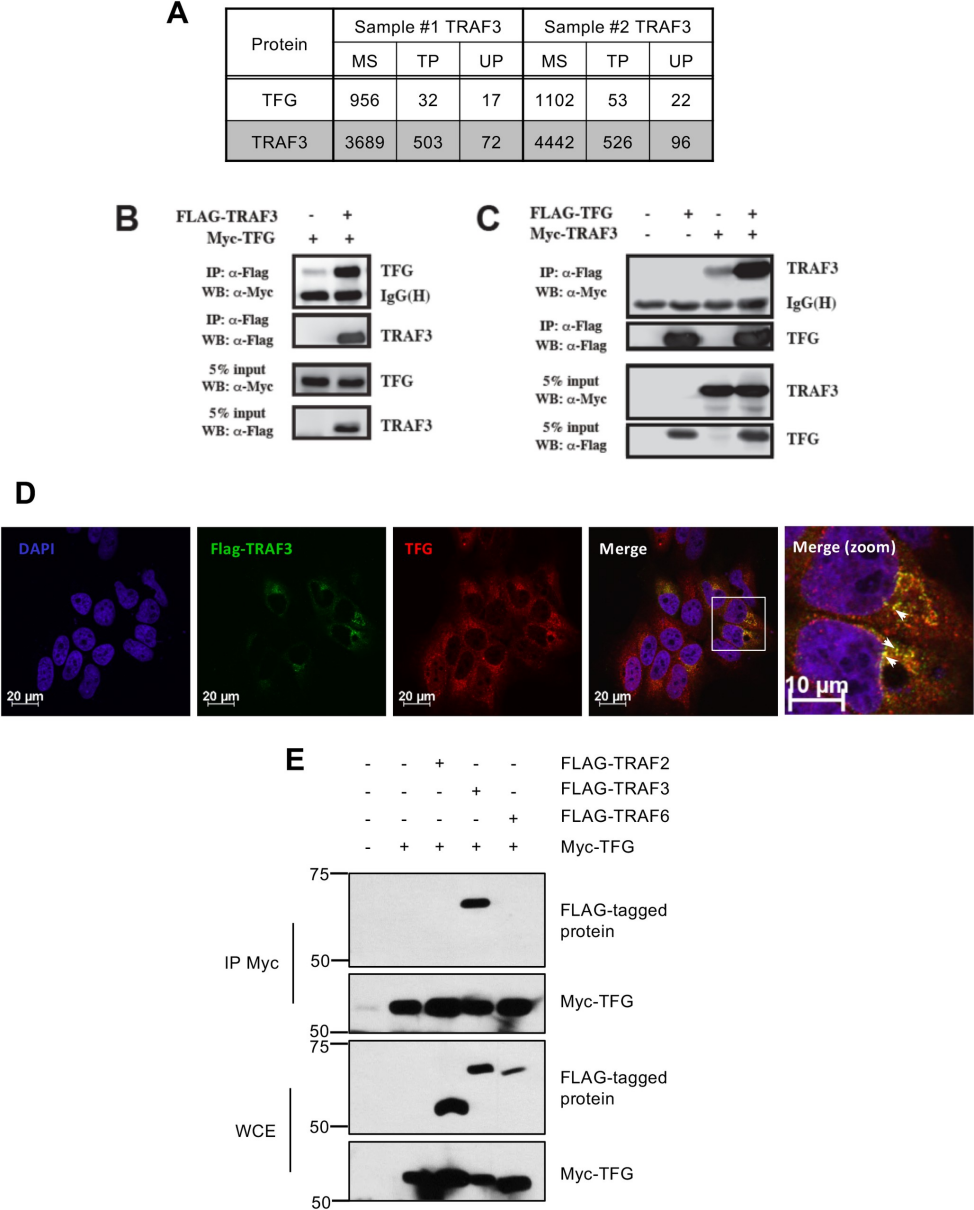
TFG interacts and colocalizes specifically with TRAF3. (A) HEK293T cells were stably transfected with pcDNA3-FLAG-TRAF3 or pcDNA3-FLAG alone. After G418 selection, cells were lysed and subjected to AP/MS as described here [48]. Data for TFG, which was undetected in control experiments, represent previously unpublished information from two biological replicates. MS; mascot score, TP; average total number of peptides (spectral counts) identified, UP; number of unique peptides observed. (B-C) HEK293T cells were transiently transfected with empty vector (-) or with vectors encoding FLAG-tagged TRAF3 (FLAG-TRAF3) together with Myc-tagged TFG (Myc-TFG) or FLAG-tagged TFG (FLAG-TFG) together with Myc-tagged TRAF3 (Myc-TRAF3). FLAG-tagged proteins were immunoprecipitated (IP) and analysed with anti-FLAG (M2) or anti-Myc (A-14) antibodies. Whole cell extracts (WCE) were also analyzed in parallel. Data represents representative results from at least 3 independent experiments. (D) HeLa cells were transfected with FLAG-TRAF3 encoding plasmids before being fixed, permeabilized and immunostained with anti-FLAG (M2) or anti-TFG antibodies. Nuclei were labeled with DAPI. Cells were then visualized by confocal microscopy. Scaling bars represent identified length. All images for all panels were representative of at least two independent experiments in which cells were examined and displayed similar staining. (E) HEK293T cells were transfected with empty vector (-) or with vectors encoding Myc-tagged TFG (Myc-TFG) together with FLAG-tagged TRAF2, TRAF3 or TRAF6. Myc-TFG was immunoprecipitated and subjected to immunoblot analysis using anti-Myc (A-14) and anti-FLAG (M2) antibodies. WCE were also analyzed in parallel. Data represents representative results from at least three independent experiments.

Our group previously identified probable TRAF3-interacting proteins that could be implicated in antiviral innate immunity through a functional proteomics approach based on FLAG affinity purification and mass spectrometry analysis (AP-MS), and functionally characterized interactions of TRAF3 with Sec16A and USO1 (also known as p115), components of the ER-to-Golgi vesicular pathway [48]. This screen identified TFG as one of the most prevalent protein in TRAF3-immunocomplexes (Fig 1A). Therefore, to confirm the physical interaction between TRAF3 and TFG, we performed co-immunoprecipitation experiments and we found that ectopically expressed tagged versions of TFG were part of immunocomplexes containing TRAF3 in the human embryonic kidney (HEK) 293T cells (Fig 1B and 1C). To further substantiate the subcellular proximity between these proteins in a context in which cell integrity is not altered, confocal microscopy experiments were done. Ectopically expressed or endogenous TFG indeed colocalized with FLAG-TRAF3 in HeLa cells (Fig 1D and S1 Fig).

**Fig 2:**
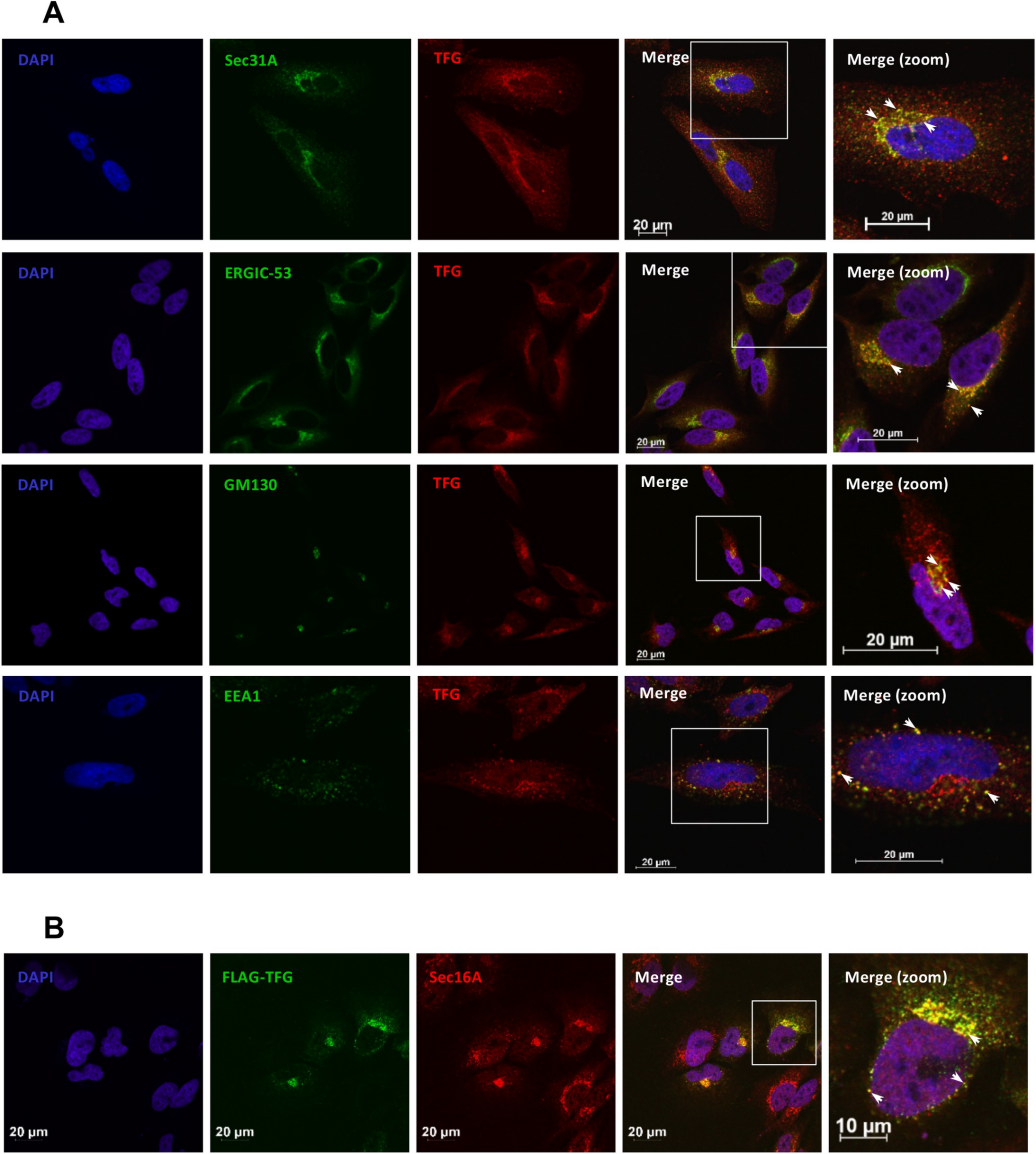
TFG accumulates within ER-to-Golgi compartments. (A) HeLa cells were immunostained for endogenous TFG along with different markers of the ER-to-Golgi associated compartments including Sec31A, ERGIC-53, GM130 and EEA1. Nuclei were labeled with DAPI. Cells were then visualized by confocal microscopy. Scaling bars represent identified length. (B) HeLa cells were transfected with FLAG-TFG encoding plasmids before being fixed, permeabilized, and immunostained with anti-FLAG (M2) or anti-Sec16A antibodies. Nuclei were labeled with DAPI. Cells were then visualized by confocal microscopy. Scaling bars represent identified length. All images for all panels were representative of at least two independent experiments in which cells were examined and displayed similar staining.

The adaptor proteins from the TRAF family are known regulators of multiple receptors including TNFR, interleukin-1 receptor (IL1R), and TLRs. They are known to bridge intracellular domains of these receptors to downstream effectors in the inflammatory and innate immune signaling pathways. TRAFs contain a C-terminal TRAF domain and often share common interacting partners. Indeed, TRAF2, TRAF3 and TRAF6 were shown to interact with MAVS [19,22,24,57]. Consequently, to verify the possible binding of TFG with other members of the TRAF family, we co-immunoprecipitated ectopically expressed Myc-TFG along with FLAG-tagged TRAF2, TRAF3 or TRAF6 in HEK293T cells. Only FLAG-TRAF3 was recovered from Myc-TFG complexes thereby confirming the selectivity of their interaction (Fig 1E). Thus, TFG seems to accumulate in a perinuclear region (Fig 1D) where it can specifically interact with TRAF3. Our group previously observed that TRAF3 mainly colocalizes with markers of the ER-Exit-Sites (ERES), ER-to-Golgi intermediate compartment (ERGIC) and the cis-Golgi apparatus [48]. To define the subcellular organization of TFG, we performed a set of confocal microscopy experiment between TFG and different markers of perinuclear compartments at the endogenous level (Fig 2). TFG was recently shown to localize on the ER-to-Golgi compartments where it interacts with Sec16A to control the export of cargoes from the endoplasmic reticulum [51,55,58]. We were able to further substantiate these observations by co-labeling HeLa cells with polyclonal α-TFG antibodies and monoclonal antibodies directed against endogenous markers for sites of COPII vesicle formation on the ERES, namely Sec16A and Sec31A, but also with ERGIC-53, which accumulates on ERGIC (Fig 2A and 2B and S2 Fig) [59–61]. Additionally, a fraction of native TFG is also found to overlap with the cis-Golgi marker GM130 and the early endosome marker EEA1 (Fig 2A and S2 Fig) [62,63].

### TFG is essential for the formation of the MAVS-TRAF3-TBK1 complex and activation of TBK1 upon RLR activation

**Fig 3:**
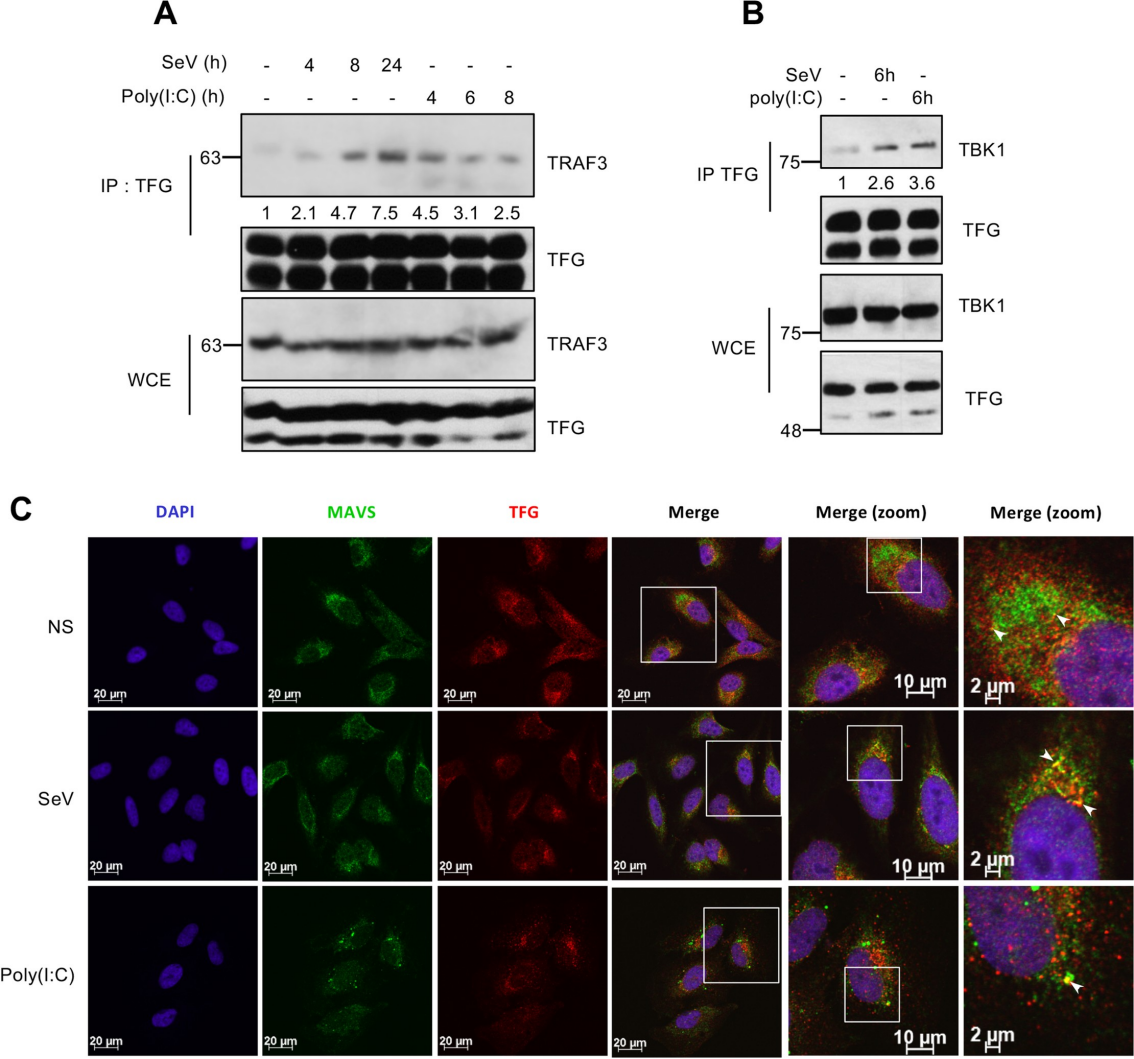
TFG is part of MAVS-TRAF3-TBK1 molecular complex upon activation of intracellular RNA sensors. (A, B) Whole cell extracts (WCE) were prepared from HeLa cells subjected to SeV infection or transfected with Poly(I:C) for indicated times and were then immunoprecipitated using antibodies directed against endogenous TFG before being immunoblotted for the presence of endogenous TRAF3 (A) and TBK1 (B). WCE were also immunoblotted in parallel. Immunoblots shown are from a single experiment and are representative of at least three independent experiments. Input-normalized TRAF3 and TBK1 densitometric signal is shown below the blot. (C) HeLa cells were either infected by SeV for 4h or transfected with Poly(I:C) for 4h before being fixed, permeabilized and immunostained with anti-MAVS or anti-TFG antibodies. Nuclei were labeled with DAPI. Cells were then visualized by confocal microscopy. Scaling bars represent identified length. White arrows represent sites of close proximity (NS) or colocalization (SeV and Poly(I:C)) between MAVS and TFG. All images for all panels were representative of two (Poly(I:C) or three (SeV) independent experiments in which cells were examined and displayed similar staining.

As aforementioned, TRAF3 is an essential player of the type I IFN arm of the RLR-dependant antiviral response. Upon viral infection, together with TBK1, TRAF3 transits from the perinuclear region onto MAVS-containing supramolecular complexes and promotes signaling events leading to TBK1 activation [38,48]. Exogenous overexpression of tagged-proteins can overwhelm the trafficking machinery, potentially affecting subcellular dispersion of proteins and leading to their mislocalization [64]. Therefore, we also performed co-immunoprecipitation of the endogenous proteins to further characterize the dynamics of interaction between TFG and TRAF3 upon viral infection. Interestingly, upon SeV infection or stimulation with Poly(I:C), TRAF3 recruitment to TFG-containing immunocomplexes was further increased compared to that in control cells, plateauing at 4h for poly(I:C) transfection and increasing between 8 to 24 h post infection with SeV (Fig 3A and S3A Fig). Additionally, the TRAF3 substrate TBK1 was also recruited to TFG upon viral infection or stimulation with Poly(I:C) (Fig 3B). Previous studies showed that infection of HEK293T cells with SeV enhanced the interaction of TRAF3 with both its upstream regulator MAVS and downstream effector TBK1 [65,66]. We next sought to determine if TFG could be part of the TRAF3-containing signaling platform near MAVS. To do so, we performed confocal microscopy experiments between endogenous TFG and MAVS to assess their possible proximity within the cell. Interestingly, a pool of TFG appeared to be in close proximity to MAVS protein, probably on an interface between the ER and mitochondria, in uninfected cells. This close proximity intensifies in certain points early after SeV infection and poly(I:C) stimulation with a subset of TFG localizing with MAVS (Fig 3C and S4 Fig). Of note, upon stimulation with poly(I:C), a potent ligand for RLR, MAVS polymerized into dense punctae compared to mock infected cells (Fig 3C) [67]. TFG also appeared to be loaded on a subset of these punctae, thereby suggesting that TFG is indeed part of the MAVS signalosome complex. In summary, our data suggest that TFG localizes with COPII vesicle markers, which transit from the ERES to the ERGIC en route to cis-Golgi compartments [68], but also with the mitochondria where it could regulate antiviral signalling events owing to its ability to interact with TBK1 and TRAF3.

**Fig 4:**
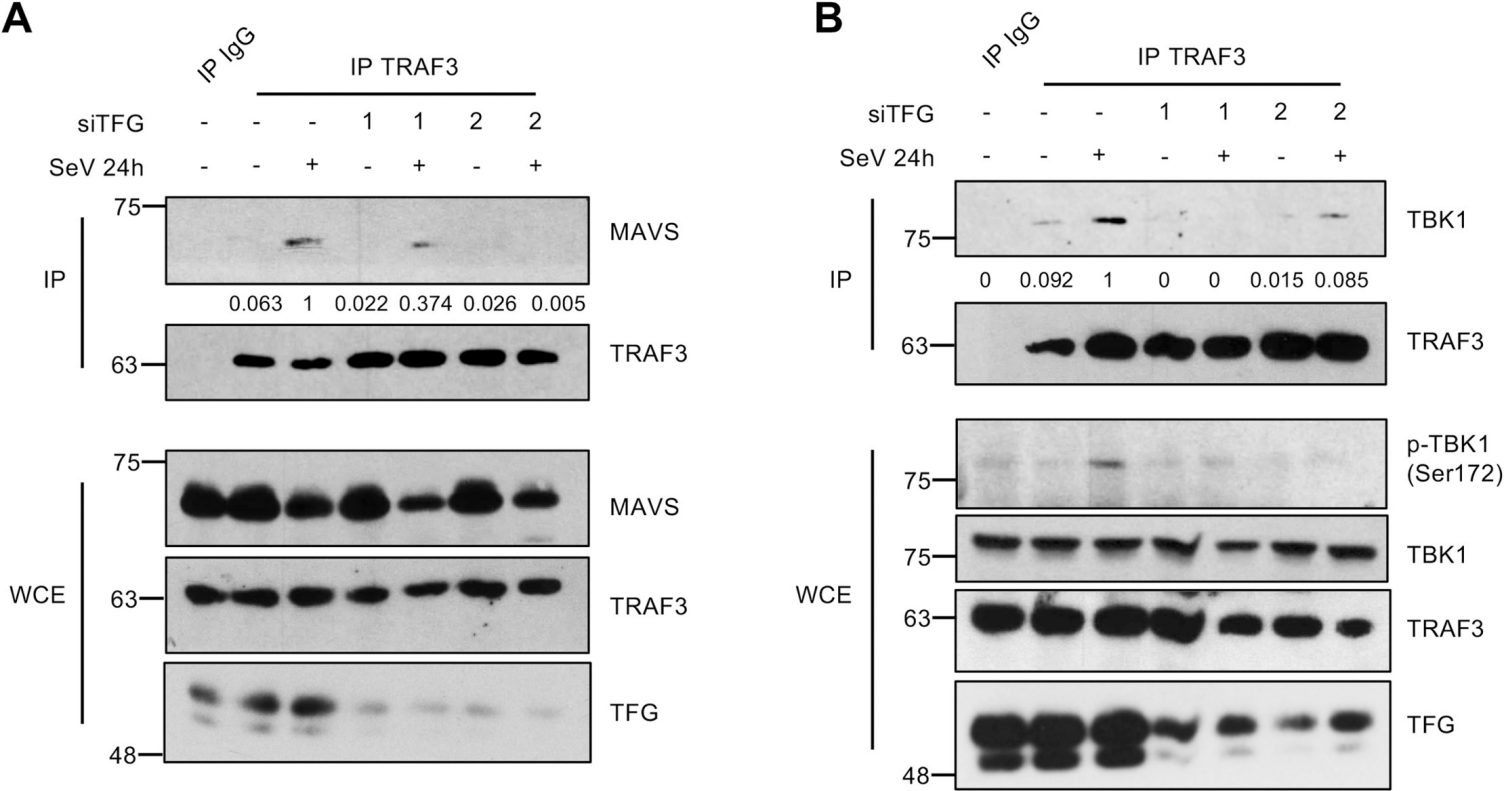
TFG is required for the formation of MAVS-TRAF3-TBK1 complex upon cytosolic RNA sensor activation. (A-B) Co-immunoprecipitation experiments were carried using HEK293T previously transfected with indicated siRNA followed by SeV infection. TRAF3 was immunoprecipitated from the prepared whole cell extracts (WCE) using antibodies directed against endogenous TRAF3 (anti-TRAF3 H-20) or isotype control antibodies (IgG) before being immunoblotted for the presence of endogenous MAVS and TBK1. WCE were also immunoblotted with the indicated antibodies. Immunoblots shown are from a single experiment and are representative of two independent experiments. Input-normalized densitometric signal of MAVS and TBK1 was divided by Input-normalized TRAF3 signal and shown below the blot.

We next tested whether TFG could be involved in the organization of MAVS signalosome networks upon viral infection. To this end, we examined the ability of TRAF3 to interact with both MAVS and TBK1 upon viral infection in HEK293T cells in which endogenous TFG was knocked down with a TFG-specific short interfering RNA (siRNA, siTFG). Cells transfected and expressing a non-targeting control siRNA (siNT) served as control. Depletion of endogenous TFG by two unrelated siRNAs reduced the extent of recruitment of TRAF3 to MAVS and TBK1 triggered by SeV infection (Fig 4A and 4B). Moreover, consistent with previous reports showing the recruitment of TRAF3 to MAVS as an important process leading to downstream signaling, the silencing of TFG also blunted the activating transautophosphorylation of TBK1 on Ser172 (p-TBK1 Ser172) normally observed upon viral infection (Fig 4B).

**Fig 5:**
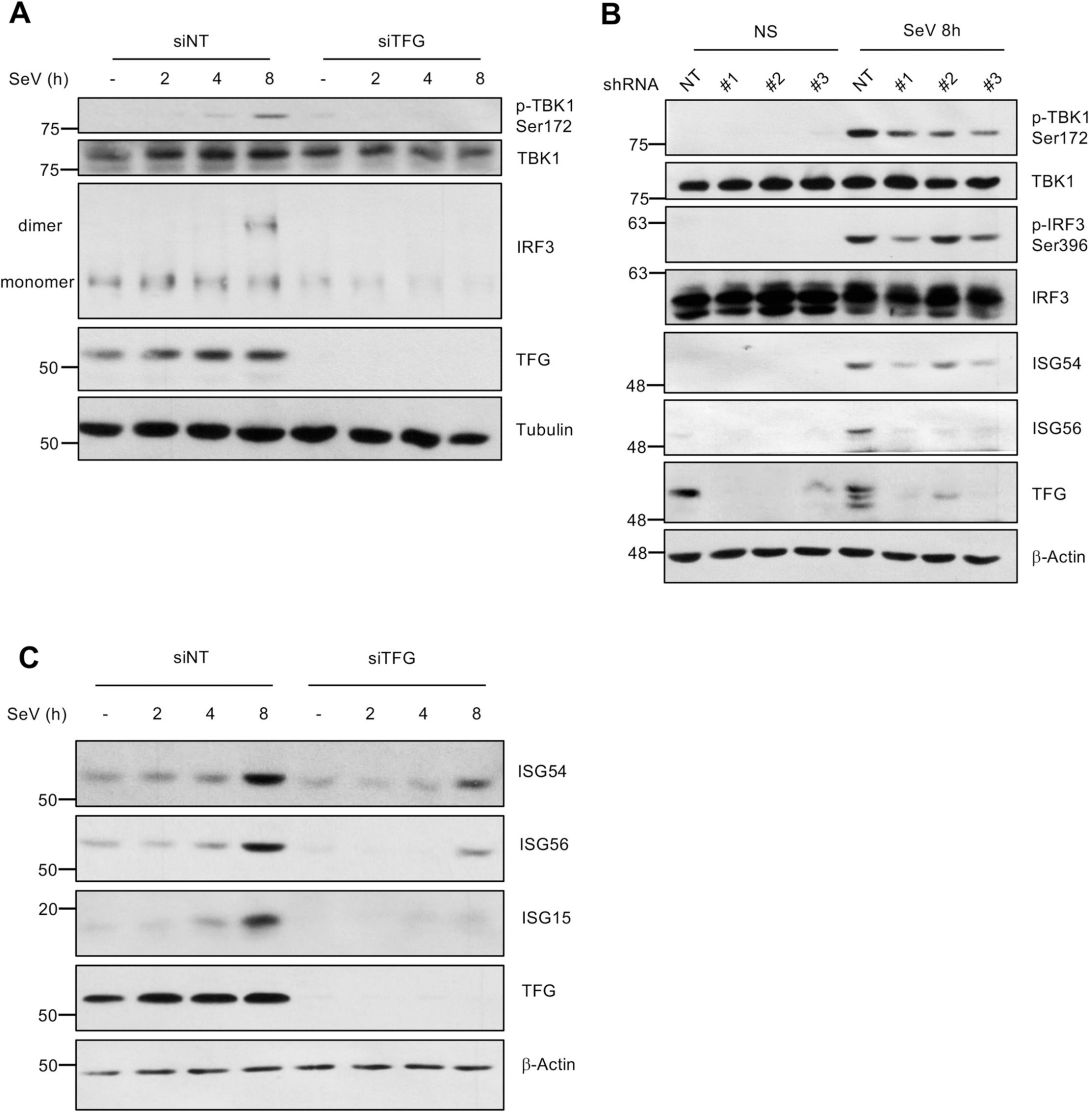
TFG is required for the activation of TBK1 and downstream signalling upon cytosolic RNA sensor activation. (A) HeLa cells previously treated with non-targeting siRNA (-) or siRNA targeting TFG (siTFG #1) were left uninfected or infected with SeV for indicated time. Whole cell extracts (WCE) were used in immunoblot analysis with indicated antibodies. The same WCE were used in native-page under non-denaturing conditions to evaluate IRF3 dimerization. (B) MRC-5 fibroblasts were infected with different lentiviral vectors encoding different TFG-targeting shRNA (shTFG #1, 2 or 3) or a nontargeting (NT) control shRNA (shNT) and then subjected to puromycin selection as described in Materials and Methods. Cells were then left uninfected or infected with SeV for the times indicated. WCE were harvested and used in immunoblot analysis with indicated antibodies. β-actin was used as a loading control. These results are representative of at least three independent experiments with similar results. (C) WCE generated in (A) were used in immunoblot analysis with indicated antibodies. β-actin was used as a loading control. These results are representative of at least three independent experiments with similar results.

**Fig 6:**
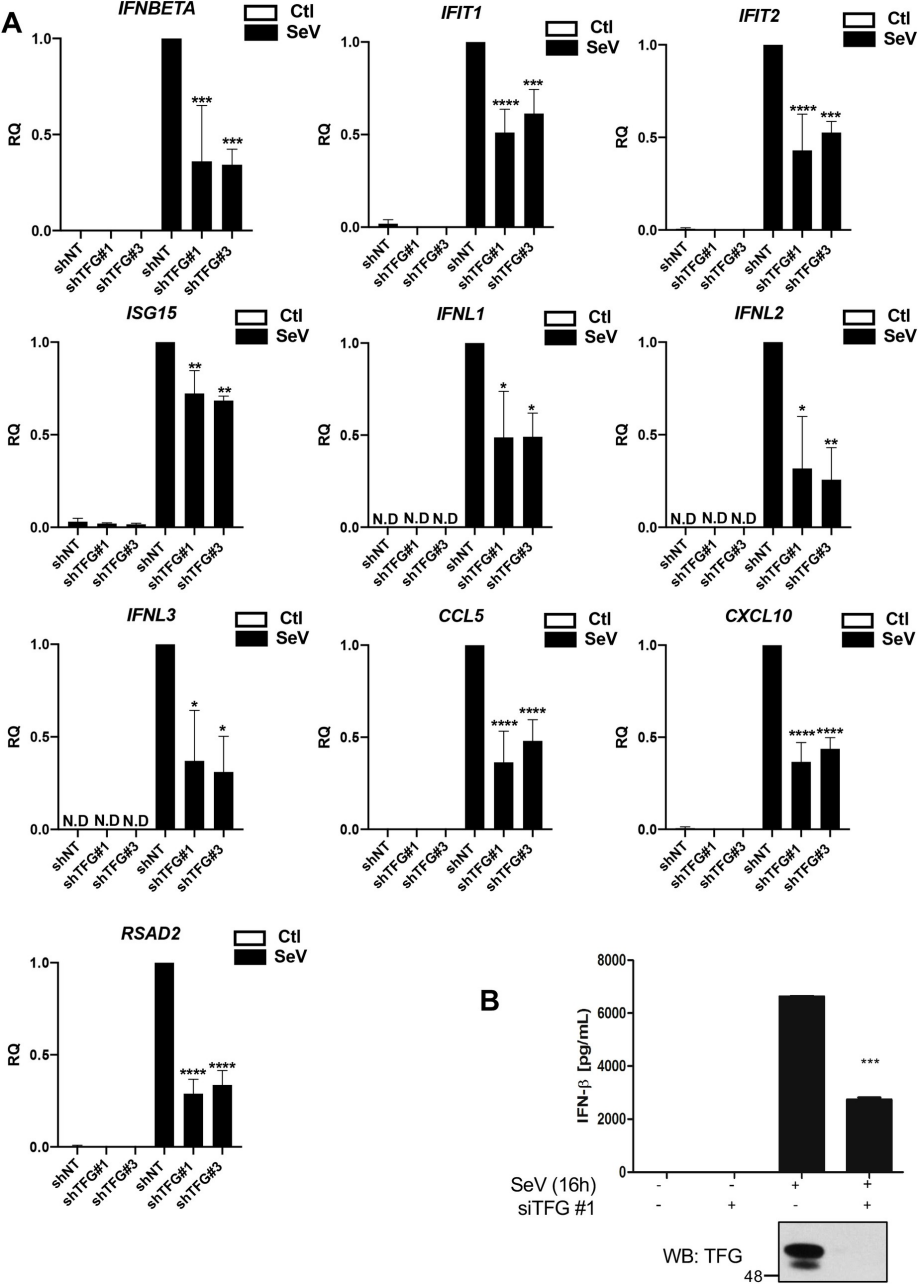
TFG is required for optimal production of ISGs and type I IFN secretion upon viral infection. (A) MRC-5 fibroblasts were infected with different lentiviral vectors encoding different TFG-targeting shRNA (shTFG #1, 2 or 3) or a nontargeting (NT) control shRNA (shNT) and then subjected to puromycin selection as described in Materials and Methods. Cells were then left uninfected or infected with SeV for the indicated times. RNA was extracted and analyzed by RT-qPCR for indicated gene expression. Mean values and SD of biological triplicates are shown * Significantly below the induction response; * P<0.05, ** P<0.01, *** P<0.001, **** P<0.0001. RQ, relative quantification. N.D, Not detected. (B) HeLa cells previously treated with non-targeting siRNA (-) or siRNA targeting TFG (siTFG #1) were left uninfected or infected with SeV for indicated time. Supernatants were collected post-infection and analyzed for IFN-β by ELISA. Mean values and SD of biological triplicates are shown (*** P-value < 0.001).

As mentioned previously, IRF3 is a critical mediator of type I interferons response following viral infection. In fact, TBK1 mediates the phosphorylation-dependent dimerization of IRF3, allowing it to properly bind with response elements sites within IFN promoters. To evaluate more precisely the role of TFG in downstream signaling of TRAF3, we followed post-translational modifications of TBK1 and IRF3 as biomarkers of TBK1 activity. TFG knockdown led to a decrease of SeV-induced phosphorylation of TBK1 and homodimerization of IRF3 compared to siNT treated HeLa cells (Fig 5A and S3B Fig). To further confirm the involvement of TFG in regulating TBK1 and IRF3 activation in a primary cell type, we used primary MRC-5 fibroblasts in which endogenous TFG was selectively knocked down using a short hairpin RNA (shRNA) approach (shTFG). Importantly, altering the expression of TFG by three shRNA constructs led to a decrease in the SeV-induced phosphorylation of both TBK1 (Ser172) and its substrate IRF3 (Ser396) (Fig 5B and S3C Fig). IRF3 plays a significant role in host survival following viral infection [69,70]. Indeed, besides its essential role in the induction of IFN-β, IRF3 contributes to the expression of different antiviral proteins, including ISG15, ISG54, ISG56 by binding to interferons-sensitive response elements (ISRE) present within the promoter region of these genes [71–73]. We further substantiated the function of TFG by measuring the expression of these IRF3-regulated antiviral ISGs in infected cells via western blot analysis. Infection of HeLa cells and primary MRC-5 fibroblasts with SeV led to the TFG-dependent production of detectable amounts of ISG15, ISG54 and ISG56 proteins (Fig 5B and 5C). Consistently, the induction of IRF3-regulated genes *IFNB1*, *IFIT2* (ISG54), *IFIT1* (ISG56), *ISG15*, *RSAD2* (Viperin), *CXCL10*, *IFNL1*, *IFNL2* and *IFNL3* in response to SeV infection was decreased following the knockdown of TFG by two different shRNA construct in primary MRC-5 fibroblasts (Fig 6A). To corroborate this finding, IFN-β production was evaluated by ELISA. Upon virus infection, siRNA-mediated silencing of TFG in HeLa cells markedly decreased production and secretion of IFN-β (Fig 6B). Thus, TFG expression in cells is important for the production of antiviral proteins following viral infection with an RNA virus. We observed no effect on the phosphorylation of TBK1 in TFG-silenced cells in response to DNA sensor agonists, including poly dA:dT, ISD, cGAMP and VACV-70 (S5 Fig).

### TFG is essential for the phosphorylation of mTOR on Ser2159, a putative TBK1 phosphoacceptor site

**Fig 7:**
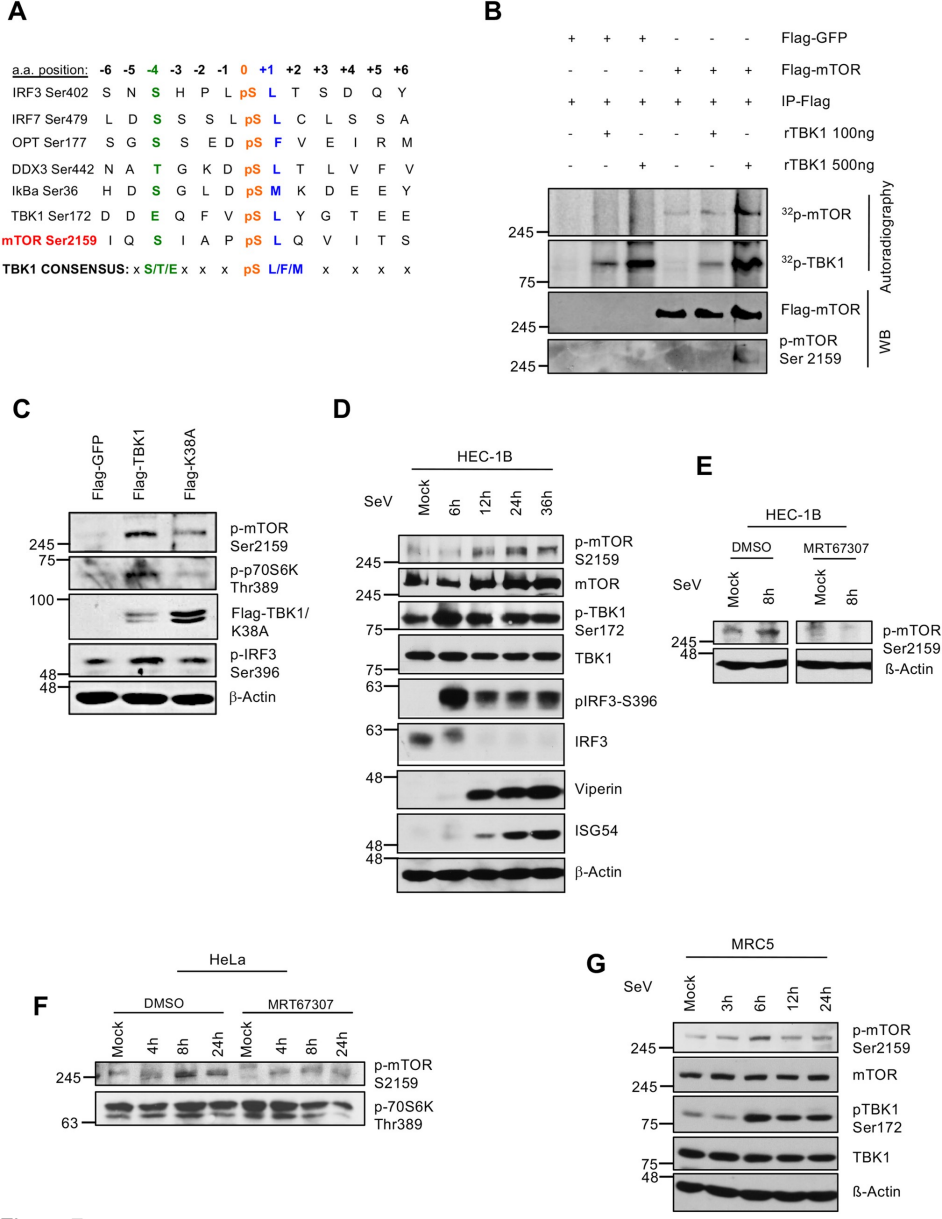
TBK1-dependent phosphorylation of mTOR on Ser2159 during viral infection. (A) Phosphorylation and sequence alignment of different substrates of TBK1 reveal a conserved consensus site. The TBK1 phosphorylation consensus sequence is composed of a central serine that is surrounded by a hydrophobic residue (L/F/M) at the +1 position relative to the phosphorylation site and a polar uncharged side chain (S/T) at the -4 position. TBK1 autophosphorylation at Ser172 closely follows this consensus except for the -4 residue which is represented by a negatively charged side chain. (B) FLAG-GFP and FLAG-mTOR were immunoprecipitated (IP) with FLAG M2 antibody from transfected HEK 293T cells. *In vitro* kinase assay was conducted by adding indicated amount of recombinant TBK1 and radiolabeled ATP and incubating at 30°C for 30 min followed by detection with autoradiography and immunoblot using indicated antibodies. Data represents representative results from at least 2 independent experiments. (C) HeLa cells were transfected with FLAG- GFP, FLAG-TBK1 and FLAG-TBK1(K38A) mutant. Next day, the media was changed with serum free media for 30h and whole cell extracts (WCE) were subjected to immunoblot analysis with indicated antibodies. Data represents representative results from at least 2 independent experiments. (D, G) HEC-1-B (D), and MRC-5 (G) cells were serum starved for 30h and then left uninfected or infected with SeV for indicated time. WCE were subjected to immunoblot analysis with indicated antibodies. (E, F) HEC-1-B (E), and HeLa (F) cells were serum starved for 30h and then incubated with DMSO and a specific TBK1 inhibitor (MRT67307; 2μM) for 2h before infection with SeV for 16h under the continuous presence of DMSO or inhibitor. WCE were subjected to immunoblot analysis with indicated antibodies. Data represents representative results from at least 2 independent experiments.

**Fig 8:**
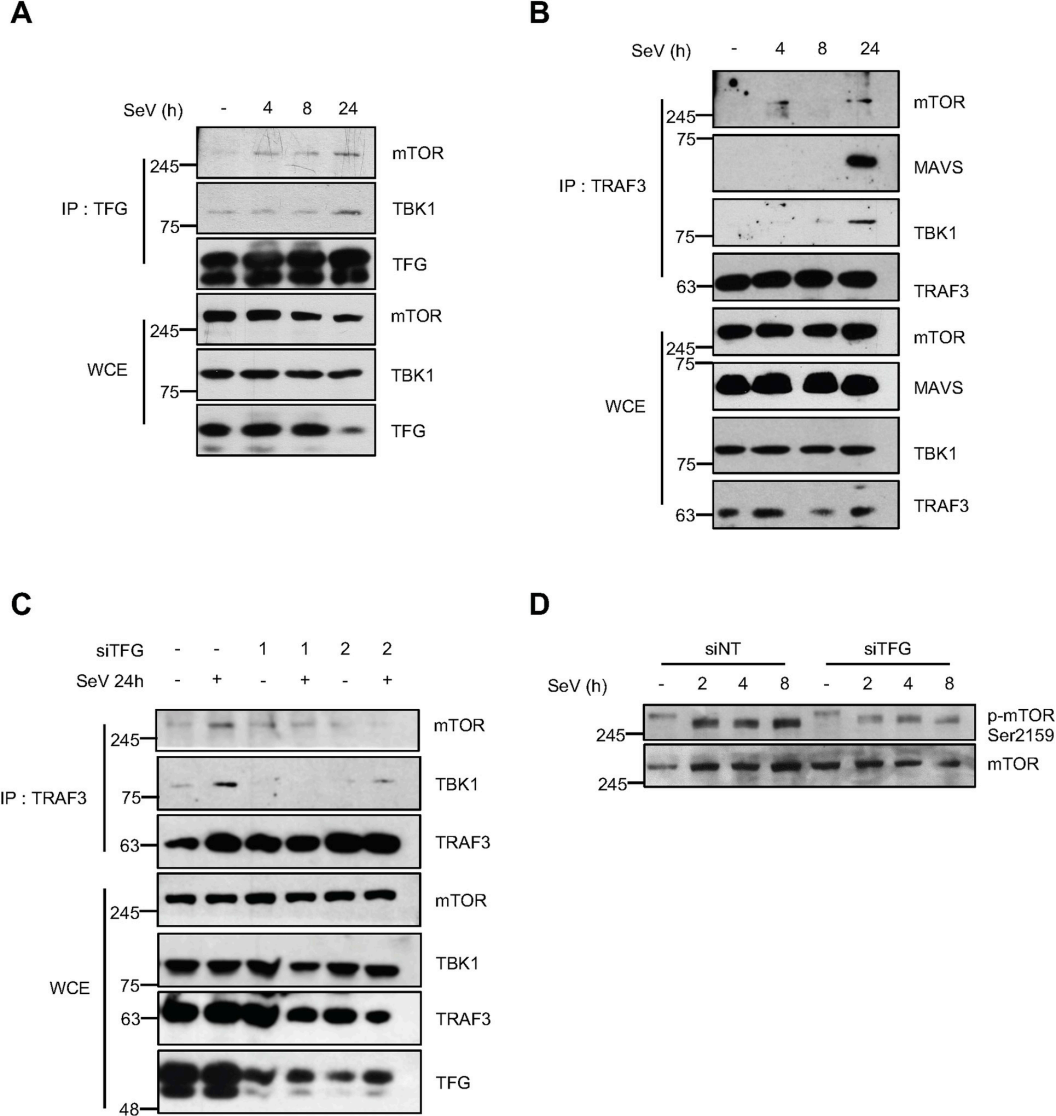
TFG is required for the proper positioning of mTOR with TBK1-TRAF3 complex as well as its phosphorylation on Ser2159, the putative TBK1 phosphoacceptor site. (A) Co-immunoprecipitation (IP) experiments from whole cell extracts (WCE) prepared from HEK293T cells infected with SeV using antibodies directed against endogenous TFG and immunoblotted for the presence of endogenous mTOR and TBK1. WCE were also immunoblotted with the indicated antibodies. Immunoblots shown are from a single experiment and are representative of three independent experiments. (B) Co-IP experiments from WCE prepared from HEK293T cells following SeV infection using antibodies directed against endogenous TRAF3 and immunoblotted for the presence of endogenous mTOR, MAVS and TBK1. WCE were also immunoblotted with the indicated antibodies. Immunoblots shown are from a single experiment and are representative of three independent experiments. (C) Co-IP experiments were carried out from HEK293T WCE previously treated with indicated siRNA followed by SeV infection using TRAF3 antibody. The same WCE were also used in immunoblot analysis with indicated antibodies. Immunoblots shown are from a single experiment and are representative of two independent experiments. (D) Following the depletion of TFG, HeLa cells were infected with SeV for the indicated time. WCE were subjected to immunoblot analysis using the indicated antibodies. N.B: Data from Fig 8C was obtained simultaneously with that for Fig 4B. It was separated for clarity of presentation. Hence, the data are the same, except for the presentation of the immunoblot for mTOR.

In addition to IRF3, TBK1 also targets many other substrates involved in antiviral innate immune response [74]. Notably, a recent study showed that the phosphorylation of mTOR on Ser2159 by TBK1 activates the mTORC1 complex, a process required for the nuclear translocation of IRF3 and the production of IFNβ upon TLR3 and TLR4 stimulation [75]. Studies have demonstrated a preferred TBK1 consensus site on known TBK1 substrates; the most striking being a preference for a hydrophobic amino acid (L or F) in the +1 position in the -S-x-x-x-pS(L/F)- amino acid sequence context [76]. Strikingly, Ser2159, in the mTOR kinase domain, perfectly fits this consensus (Fig 7A). Using *in vitro* kinase assays with recombinant TBK1 and immunoprecipitated mTOR, we document the phosphorylation of mTOR on Ser2159 (Fig 7B). Accordingly, when mTOR was co-expressed with WT Flag-TBK1 or its kinase dead version K38A, site-specific phosphorylation signal on Ser2159 was observed in WT TBK1 expressing cells which was decreased in K38A cells. Activation of mTORC1 signaling module was evident in TBK1 expressing cells through an increase in phosphorylation of p70S6K Thr389, a known mTORC1 substrate (Fig 7C). Since it was still unknown whether this novel paradigm in antiviral signaling occurs upon RLR engagement, we next addressed the phosphorylation of mTOR on Ser2159 in cells infected with SeV. To confirm that this modification of mTOR occurs independently of the activation of IFN receptors and the secreted IFNα/β, we verified if this molecular event occurred in the IFN non-responsive HEC-1-B cell line [77–79]. In serum-deprived cells, SeV infection caused a sustained increase of mTOR on Ser2159 (Fig 7D). The use of the selective TBK1 ATP-competitor inhibitor MRT67307 [80] showed that the virus-induced phosphosignal was dependent on TBK1 catalytic activity (Fig 7E and 7F). Importantly, this modification of mTOR was also observed in primary fibroblasts infected with SeV (Fig 7G). To better describe molecular events underlying mTOR activation downstream of TBK1, we sought to determine how TBK1 engages with mTOR during viral infection. Selective activation of mTOR at the lysosomes is obviously a possibility due to its lysosomal localization [81]. However, recent observations rather imply that Rheb-GTPases-induced mTOR activation occurs in a membrane-rich environment from multiple organelles [82]. Since TFG localizes within the ER-to-Golgi compartments (Fig 2), we next verified if mTOR could exist in complex with TFG. Interestingly, endogenous mTOR and TBK1 exist in TFG and TRAF3 immunocomplexes and their interaction increases following SeV infection in HEK293T cells (Fig 8A and 8B). Furthermore, the ability of TRAF3 to associate with mTOR, and TBK1 following SeV infection as well as the virally induced Ser 2159 phosphosignal require the presence of TFG (Fig 8C and 8D).

### mTORC1 activation controls ISG expression without affecting the phosphorylation, dimerization and nuclear accumulation of IRF3

**Fig 9:**
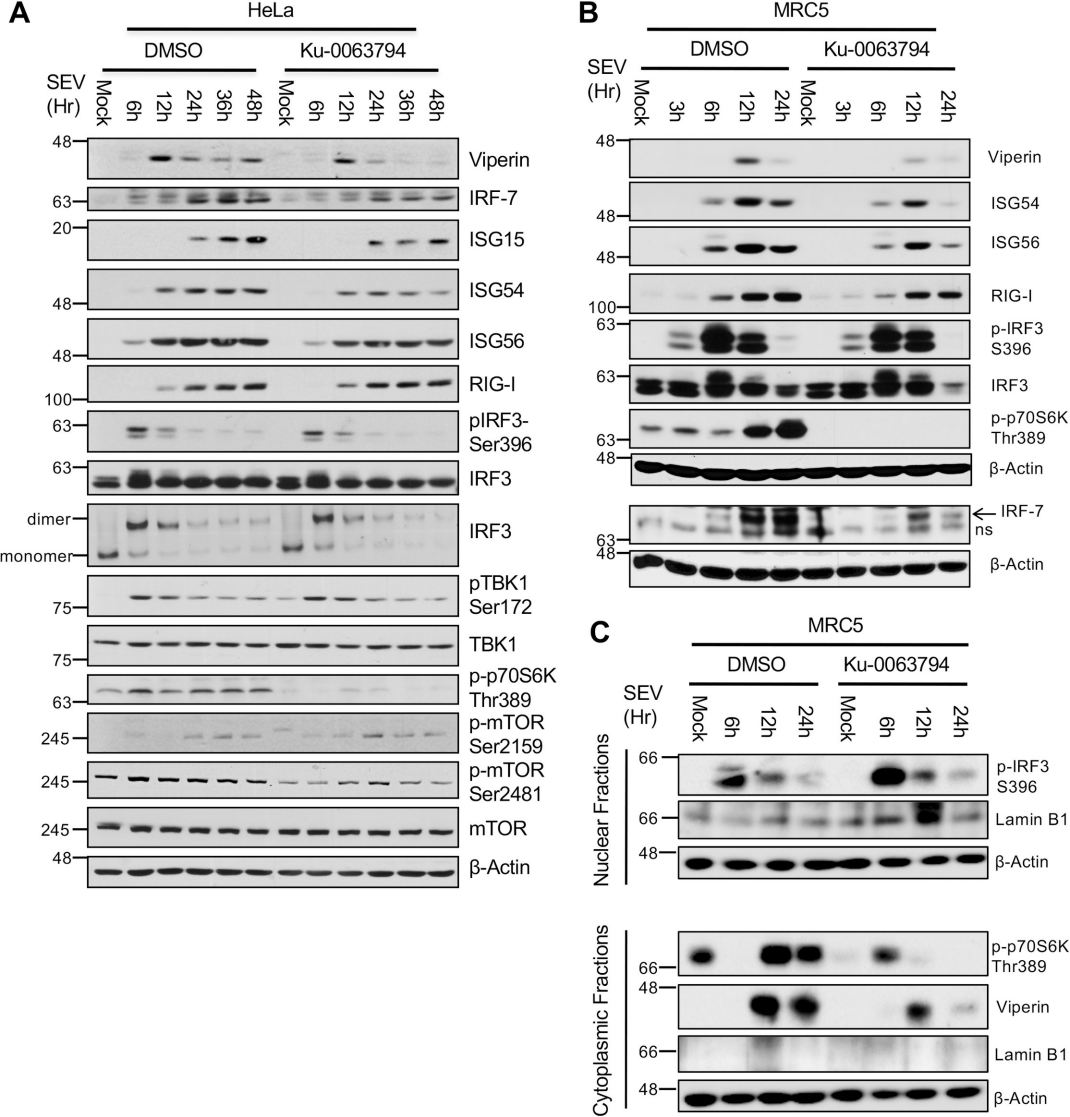
Implication of mTOR in the induction of selected sets of Interferon-Stimulated Genes (ISGs) during RLR signaling. Serum starved HeLa cells (A) or primary MRC5 fibroblasts (B) were pretreated with 0.5 μM Ku-0063794, a highly selective mTOR inhibitor, or vehicle for 30 minutes and then left uninfected or infected with SeV (200 HAU/ml) for the indicated times in the continuous presence of the drug. Whole cell extracts (WCE) were subjected to immunoblot analysis using the indicated antibodies. (C) Serum starved primary MRC5 fibroblasts were treated as described above. Crude nuclear and cytoplasmic fractions were prepared and subjected to immunoblot analysis using the indicated antibodies. Immunoblots shown are from a single experiment and are representative of three independent experiments.

mTOR phosphorylation on Ser2159 induces the activation of mTORC1 and promotes mTORC1-associated mTOR S2481 autophosphorylation [83]. In the RLR pathway, we also document mTORC1 activation in SeV-infected cells, as measured through the increased phosphorylation of mTOR on Ser2481 and/or phosphorylation of p70S6K on Thr389 (Figs 7F, 9A and 9B), molecular events that are blunted by the use of Ku-0063794, a highly potent and selective mTOR inhibitor. However, whereas the use of the inhibitor affected the expression of antiviral proteins under the direct control of IRF3 (ISG54, ISG56, Viperin) and type I/III IFNs (IRF7) other ISGs such as RIG-I were less affected. The use of the mTOR inhibitor neither change the induction of the respective mRNA levels (S6 Fig) nor affected the phosphorylation-dependent dimerization of IRF3 (Fig 9A and 9B). Moreover, the use of the mTOR inhibitor Ku-0063794 or the mTORC1 inhibitor rapamycin did not affect the ability of IRF3 to accumulate into the nuclear compartment following the infection of primary fibroblasts with SeV (Fig 9C and S7A Fig). Furthermore, whereas ectopically expressed mTOR synergized with the constitutively active version of RIG-I to induce the activation of the mTORC1 substrate p70S6K (S7B Fig, compare lanes 7 and 9), the mTOR S2159A mutant showed a dominant-negative effect (compare lanes 7–8 and lanes 9–10). Under these conditions, the nuclear accumulation of IRF3 was not affected following activation of the cGAS or RIG-I antiviral signaling pathways.

Altogether our data suggest that upon RIG-I activation, TFG acts as a signaling hub through which TRAF3-associated TBK1 leads to the phosphorylation of not only IRF3 but also mTOR on Ser2159 which leads to mTORC1 activation and a proper antiviral response.

### Silencing of TFG expression compromises the establishment of an antiviral state during virus infection

**Fig 10:**
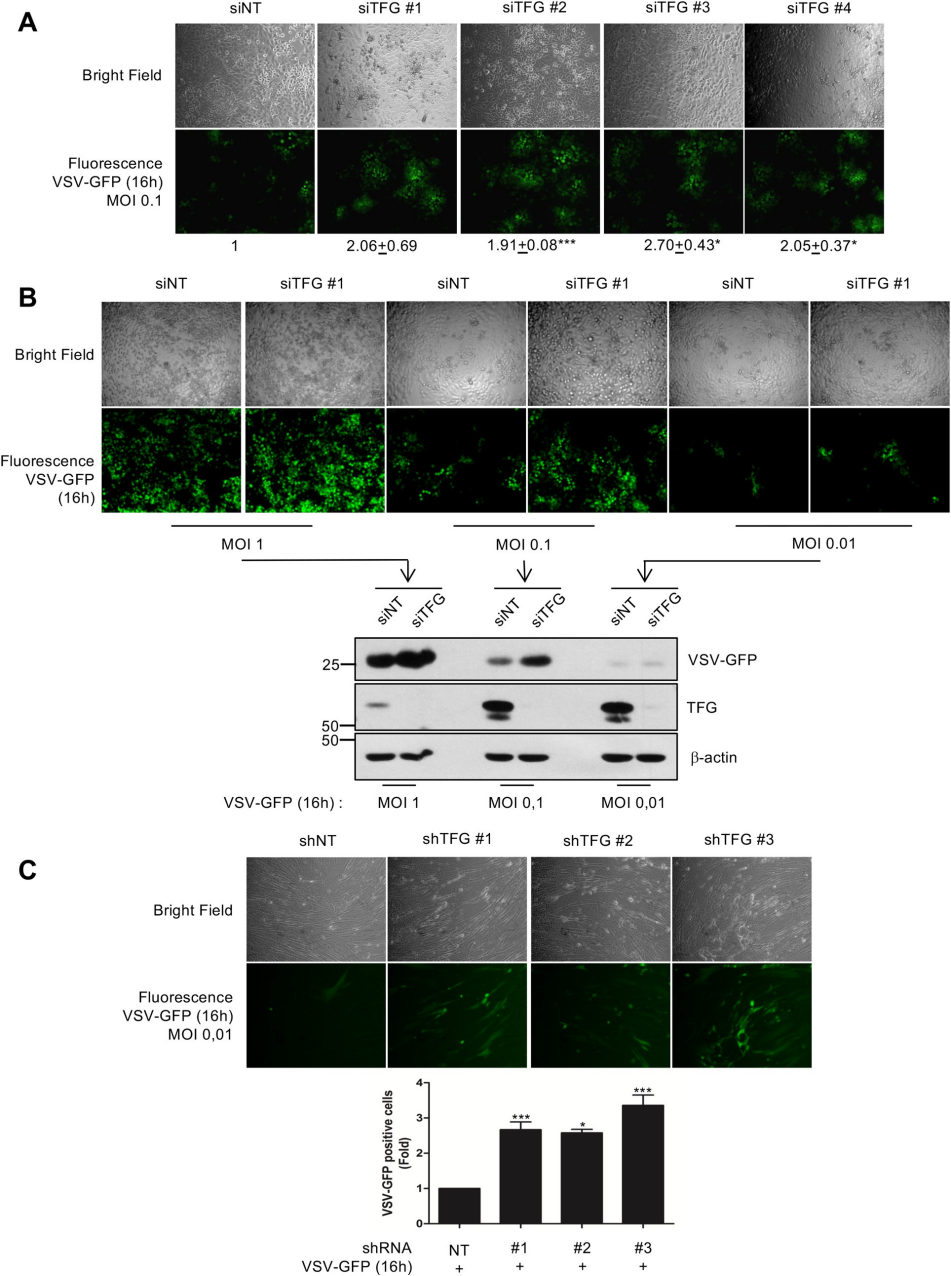
Knockdown of TFG increases viral replication and dissemination. (A) HeLa cells were treated with siNT or with four different siTFG constructs. Cells were then infected with VSV-GFP for 16h at an MOI of 0.1 and monolayers were analysed with an inverted fluorescence microscope. Observation from one experiment out of three experiments is shown. Quantification of fluorescence from 3 independent biological replicates is shown below the images. Mean values and SD of pooled data are shown (* P-value < 0.05; *** P-value < 0.001). (B) HeLa cells were treated with siNT or with siTFG #1 before being infected with VSV-GFP for 16h at indicated MOI. Monolayers were analyzed by fluorescence microscopy before whole cell extracts (WCE) were prepared. WCE were then immunoblotted with indicated antibodies. (C) MRC-5 fibroblasts were infected with different lentiviral vectors encoding different TFG-targeting shRNA (shTFG #1, 2 or 3) or a nontargeting (NT) control shRNA (shNT) and then subjected to puromycin selection. Cells were then infected with VSV-GFP for 16h at an MOI of 0.01. and monolayers were analysed with an inverted microscope. Then, the extent of VSV-GFP infection was further analysed by quantifying GFP-positive cells by flow cytometry. Data were pooled from three independent experiments and are expressed relative to their cognate shNT control from each experiment to account for day-to-day variation. Mean values and SD of pooled data are shown (* P-value < 0.05; *** P-value < 0.001).

We next determined the importance of TFG expression on the replication of VSV, which is sensed by RIG-I [14]. In fact, by using a functional antiviral assay involving a modified version of VSV expressing a GFP-tagged protein (VSV-GFP), it is possible to monitor the extent of viral replication and infection. HeLa cells in which TFG was silenced using four different siRNA exhibited substantially enhanced VSV-GFP infection rates compared to those of cells expressing siNT, as shown in fluorescence microscopy (Fig 10A). Using one of the siRNA duplexes, the observed decrease in the antiviral response in fluorescence microscopy or semi-quantitative western blot analysis was reproduced using multiple multiplicity of infection (MOI) (Fig 10B). Similarly, primary MRC-5 fibroblasts expressing TFG-targeting shRNAs were more sensitive to VSV challenge (Fig 10C). In summary, these loss-of-function experiments demonstrate that TFG expression is important for an adequate antiviral response through its interaction with different players of the antiviral response.

Together, these results suggest that TFG sustains RLR-signaling pathways by enabling an efficient organization of important mediators that result in the activation of TBK1 following the activation of MAVS. TFG is further required for downstream signaling events including phosphorylation of mTOR and IRF3 by TBK1 ultimately resulting in the establishment of an antiviral state following RLR engagement.

## Discussion

**Fig 11:**
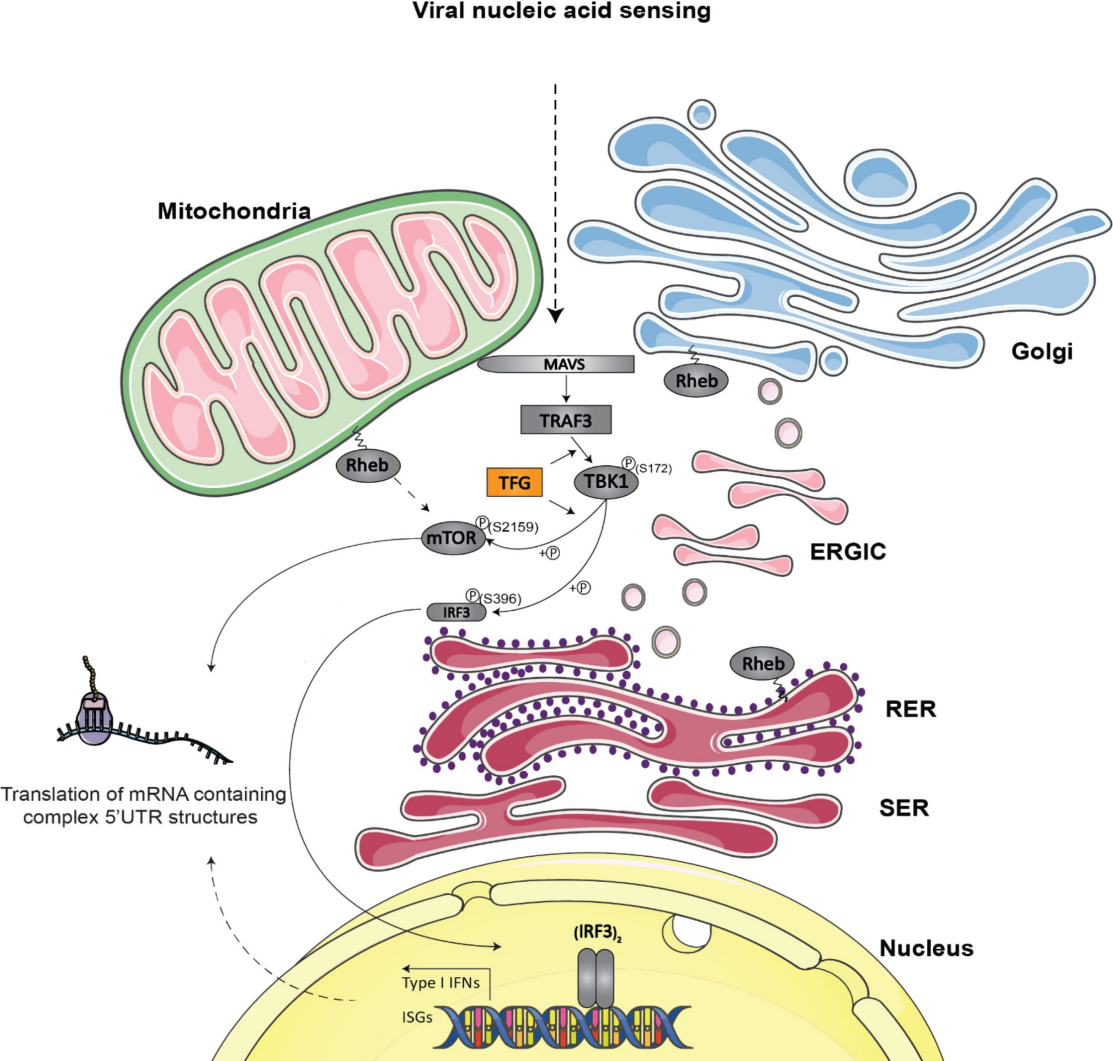
Proposed unified model representing the implication of TFG in the organization of RLR-dependent antiviral innate immunity. The ER-to-Golgi vesicular transport system serves as an organizing membrane-rich platform allowing the organization of RLR-dependent antiviral innate immunity. TFG is involved in optimizing COPII assembly at the ERES and disassembly at the ER/ERGIC interface (pink vesicles located between the RER and ERGIC). TFG thereby enables an efficient export of proteins from the ER to other organelles. Possibly through its ability to self-associate and to generate larger polymers, TFG also allows the proper positioning of essential effectors (TRAF3, TBK1) with MAVS onto an interface between mitochondria and ER-related membranes where they can functionally interact upon viral infection. These signaling events result in the phosphorylation of IRF3, its dimerization and nuclear translocation where it rapidly induces the transcription of type I IFN genes and a subset of ISGs. TFG also allows the positioning of mTOR with TRAF3-TBK1 complexes resulting in mTORC1 signaling pathway activation. The translation of a subset of ISGs mRNA is also under the control of mTORC1 pathway, which is regulated, at least in part, by TBK1. SER: smooth endoplasmic reticulum, RER: rough endoplasmic reticulum. The model was created using Servier Medical Art templates (www.servier.com) licensed under a CC BY 3.0 license (https://creativecommons.org/licenses/by/3.0/).

The RIG-I-MAVS-TRAF3 axis is recognized as a fundamental signaling pathway leading to rapid and potent antiviral host response to viral infection. An emerging paradigm proposes that RLRs are dependent on the cellular trafficking machinery to link virus sensing sites to signal transducing hub within the cell [84–86]. Whereas the regulated transport of RLRs to their cognate sorting adaptors represents a new critical checkpoint for innate immune signal transduction, much less is known concerning the events leading to the recruitment and the organization of downstream effectors. Here, we present the role of TFG as an essential component of MAVS-TRAF3-TBK1 signaling complex. Its subcellular localization allows efficient recruitment of TRAF3 to its upstream adaptor MAVS, permitting the activation of downstream kinase TBK1. Interestingly, TFG also allows the positioning of mTOR with TRAF3-TBK1 complex resulting in mTOR phosphorylation on Ser2159. Phosphorylation of IRF3 and mTOR by TBK1 is followed by the subsequent expression of type I IFNs and ISGs (Fig 11). Whereas conflicting reports regarding TFG’s role in RLR and TLR3-induced type I IFN antiviral signaling exist [87,88], our study clearly demonstrates mechanistic details of a positive molecular role of TFG in organizing antiviral responses upon RLR activation in multiple cell types.

Since VSV is a well-established IFN-sensitive virus [89,90], we confirmed the importance of TFG expression in the ability of cells to prevent VSV replication and dissemination using RNAi approaches. Confirming this notion, ELISA experiments showed the critical role of TFG in IFN-β expression/secretion. Likewise, subsets of IRF3-regulated ISGs expression following SeV infection was decreased compared to their cognate TFG-expressing controls. As such, these experiments using the RIG-I-sensed VSV and SeV reveal a potential role of TFG in RLR signaling pathways. However, the precise level at which TFG plays its role was still unknown. A previous study identified TRIM25, a known regulator of RIG-I, as a TFG-interacting partner suggesting a role of TFG in RLR signaling [87]. However, it was recently shown that Riplet, but not TRIM25, is required for endogenous RIG-I-dependent antiviral response [91]. TRIM25 can also promote K48-linked ubiquitination and degradation of target proteins, including MAVS [92–94]. Importantly, studies have shown that during RNA virus infection, expression of MAVS diminishes over time [39,92]. Paradoxically, this loss of MAVS expression appears to positively regulate antiviral signaling, as this loss immediately precedes the phosphorylation of IRF3. Thus, even though TFG interacts with TRIM25 [87], it appears to play downstream MAVS activation. Indeed, MAVS degradation pattern upon SeV infection was unchanged in TFG-silenced HeLa cells (Fig 4A). On the other hand, TFG-depletion directly affected the autophosphorylation of TBK1 within its activation loop on Ser172 and subsequent IRF3 activation, thereby proposing a role for TFG in RLR pathway downstream MAVS signaling (Figs 4B and 5). Moreover, we observed that knockdown of TFG in HeLa cells further affected IRF3 phosphorylation in conditions where MAVS degradation was avoided by pretreatment of cells with the proteasome inhibitor MG132. Altogether, these results suggest that TFG acts downstream of MAVS activation and degradation.

TBK1 functions in multiple signaling pathways, including type I IFN antiviral response and autophagy [95–97]. Growing evidence also revealed the aberrant TBK1 activity in a variety of autoimmune diseases and cancers [74,98]. As such, numerous regulatory mechanisms exist to prevent TBK1 activation in the absence of pathway stimulation [97]. The main molecular event that controls TBK1 activity is the phosphorylation of the catalytic serine residue in the activation T-loop at position 172 (Ser172) [99,100]. Existing in a dimeric assembly in a configuration limiting its transautophosphorylation [101,102], TBK1 requires K63-linked polyubiquitination modification to become active [32,103]. In RLR signaling, several E3 ligases were shown to be involved in such ubiquitination, including TRAF3 [31]. Moreover, another regulatory mechanism controlling the activation of TBK1 is the presence of scaffold proteins like TANK and NEMO bringing TBK1 and TRAF3 together onto MAVS complex [104]. However, how such movements of proteins are regulated remains unsolved. We identify TFG as being part of a molecular complex comprised of at least MAVS, TRAF3 and TBK1 and requirement of TFG in the interactions of TRAF3 with TBK1 and MAVS upon viral infection (Figs 3, 4 and 8). Mechanistically, we propose that TFG allows TRAF3 to recruit TBK1 to MAVS where, following its activation by TRAF3 [31], TBK1 contributes to the phosphorylation of MAVS and the subsequent recruitment and phosphorylation of IRF3 [105]. Interestingly, TFG was previously shown to physically interact with both TANK and NEMO, further implying a role of TFG in TBK1-mediated IRF3 activation [106]. Since NEMO is an adaptor protein promoting crosstalk between NF-κB and IRF signaling pathways [104] and given its interaction with TFG, a role of TFG in the activation of NF-κB cannot be excluded.

With accumulating evidence of the role of cellular trafficking machinery in multiple signaling pathways, it is now well-known that several organelles house and transport cellular signaling molecules [107–111] and thus, they may act as signaling hubs for crosstalk between multiple cell signaling pathways. Here, we present the crosstalk of mTOR and RLR-signaling pathways by demonstrating the requirement of TFG for the interaction of TRAF3 and TBK1 with mTOR upon viral infection. The importance of the RLR-dependent association of TRAF3 with mTOR observed here is possibly a hallmark in the ability of infected cells to produce type I IFN. In fact, it was recently discovered that mTOR and TRAF3 constitutively associate in plasmacytoid dendritic cells, the major producers of IFN-α in response to virus exposure [112]. Within the mTORC1 complex, mTOR can be phosphorylated on several activating residues, Ser2159 being one of them [83]. The phosphorylation of this phosphoacceptor site by TBK1 has recently been reported to activate mTORC1 signaling upon TLR3/TLR4 engagement and is required for the nuclear translocation of IRF3 [75]. How TBK1 engages mTOR was however not addressed. Our data demonstrate that TFG is important for TBK1-dependent activation of mTORC1 signaling following RLR engagement during SeV infection. However, further characterization of mTORC1 signaling in antiviral response proposes that it acts without affecting IRF3 phosphorylation and nuclear translocation. We propose that it is within the membrane-rich microenvironment of the secretory system (i.e. endomembranes) that TBK1, in association with TFG, interacts with and phosphorylates mTOR on Ser2159 upon RLR stimulation. Our observations suggest that TBK1 can fine tune the antiviral response by having two roles in RLR signaling. In addition to its established transcriptional role through the phosphorylation and activation of IRF3, TBK1 phosphorylates mTOR on Ser2159 leading to mTORC1 activation and canonical cap-translation of selected mRNAs such as eIF4E-sensitive transcripts IRF7 [113]. The kinetic of the phosphorylation of mTOR leading to its activation within the mTORC1 complex and the spatiotemporal regulation of such events remains unresolved. Interestingly, it was shown that mTORC1 activation could also happen at the Golgi apparatus [114], where localization of p-TBK1 has recently been reported [49]. However, the details of the movement of TBK1 between mitochondria and Golgi is not clear. As we have also observed TFG partially localized to the Golgi apparatus (Fig 2A), it is tempting to speculate that TFG plays a role in this movement and would be a subject of future studies. TBK1 is a major effector regulating autophagy and in the context of cellular growth and catabolism, it was also proposed to inhibit the mTORC1 pathway [115,116] through phosphorylation of Raptor on Ser877 [116]. Additional studies, like this one, could pave the way to understand why TBK1 activates the AKT /mTORC1 pathway under certain situations [117–119] and repress in others.

This study proposes an important role for the ER-to-Golgi vesicular transport system protein TFG in allowing the proper positioning of TRAF3 with MAVS, TBK1 and mTOR, which is required for the establishment and likely the fine regulation of antiviral signaling events. Future characterization of TFG’s implication in other PRRs-regulated pathways will undoubtedly help to appreciate the importance of the trafficking secretory pathway in innate immunity and autoimmune diseases.

## Materials and methods

### Cell culture, reagents and antibodies

Human cervical adenocarcinoma (HeLa), human embryonic kidney (HEK) 293T and primary human fetal lung fibroblast (MRC-5), and THP-1 cell lines were obtained from American Type Culture Collection (ATCC). All cell lines were maintained according to ATCC’s guidelines. HEK293T cells were cultured in high glucose Dulbecco’s Modified Eagle Medium (DMEM) (Cat# 11995–065; Invitrogen) containing 4 mM L-Glutamine (Cat# G7513, Sigma), HeLa cells were maintained in low glucose DMEM (Cat# 11885–084; Invitrogen), MRC-5 fibroblasts were cultured in Eagle’s Minimum Essential Medium (EMEM) (Cat# 11095–080; Invitrogen) complemented to 0.1 mM non-essential amino acid (Cat# 11140–050; Invitrogen) and to 1 mM sodium pyruvate (Cat# 11360–070; Invitrogen). THP-1 cells were cultured in RPMI-1640 Medium supplemented with 2-mercaptoethanol to a final concentration of 0.05 mM. All media were supplemented with 10% heat inactivated foetal bovine serum FBS-HI (Cat# 16000–044; Invitrogen).

Poly (I:C) (1μg/ml) (GE HealthCare) and the DNA sensor agonists (all from Invivogen): Poly dA:dT (2 μg/ml), ISD (2 μg/ml), cGAMP (5 μg/ml) and VACV-70 (2 μg/ml) were transfected with Lipofectamine 2000 (Invitrogen) according to manufacturer’s protocol. Polybrene and Puromycin were purchased from Sigma. Ku-0063794 and Rapamycin were from Selleck Chemicals.

Antibodies were obtained from indicated companies: anti-TFG (Cat# IMG-5901A; Novus Biologicals), anti-FLAG M2 (Cat# F3165; Sigma), polyclonal anti-FLAG (F7125; Sigma), anti-Myc 9E10 (Cat# sc-40; Santa Cruz Biotechnology), anti-Myc A-14 (Cat# sc-789; Santa Cruz Biotechnology), anti-Sec31A (Cat# 612350; BD Biosciences), anti-ERGIC-53 (Cat# 804-602-C100; Enzo Life Sciences), anti-GM130 (Cat# 610822; BD Transduction), anti-EEA1 (Cat# 610456; BD Transduction), anti-Sec16A (Cat# A300-648A; Bethyl Laboratories), anti-MAVS (Cat# ALX-804-847; Enzo Life Sciences), anti-TRAF3 G-6 (Cat# sc-6933; Santa Cruz Biotechnology), anti-TRAF3 H-20 (Cat# sc-948; Santa Cruz Biotechnology) anti-p-TBK1 Ser172 (Cat# 5483, Cell Signaling), anti-TBK1 (Cat# IMG-270A; Novus Biologicals), anti-p-IRF3 Ser396 (Cat# 4947; Cell Signaling), anti-IRF3 C-20 (Cat# sc-15991; Santa Cruz Biotechnology), anti-ISG15 (Cat# 2743; Cell Signaling), anti-ISG54 (Cat# NBP1-31164; Novus Biologicals), anti-ISG56 (Cat# NBP1-32329; Novus Biologicals), anti-Viperin (Cat# ALX-210-956, Enzo Life Sciences), anti-IRF7 (Cat# sc-9083, Santa Cruz Biotechnology), anti-β-actin (Cat# A2228; Sigma), anti-α-Tubulin (Cat# T6199; Sigma), anti-GFP (Cat# G8965-22C; Abcam), anti-p-mTOR-Ser2159 (Cat# ABS79; Millipore-Sigma), anti-mTOR (Cat# 2972; Cell Signaling), anti-p-p70S6K Thr389 (Cat# 9205; Cell Signaling), anti-mouse HRP-conjugated (Cat# 074–1806; KPL), anti-rabbit HRP-conjugated (Cat# 074–1506; KPL), anti-goat HRP-conjugated (Cat# 01-13-06; KPL), alexa 488-conjugated anti-mouse (Cat# A11001; Invitrogen) and alexa 568-conjugated anti-rabbit (Cat# A10042; Invitrogen) secondary antibodies.

### Plasmids constructs, transfections and infections

Plasmids encoding FLAG-TFG and Myc-TFG were produced from TFG cDNAs. TFG cDNAs were first amplified from the MGC bank collection and then subcloned in pTag2B (FLAG), pTag3B (Myc) and pMRX vectors. FLAG-TRAF3 and Myc-TRAF3, FLAG-TRAF2 and FLAG-TRAF6 were obtained as previously described [48]. FLAG-mTOR was a kind gift from Dr. Philippe Roux (IRIC, Université de Montréal). mTOR S2159A was produced using site-directed mutagenesis. pCDA3.1-MYC-delta RIG-I (encoding the first 128 a.a. of RIG-I; a constitutively active version of RIG-I) was received from Dr. Rongtuan Lin (McGill University). pCDNA3.1 FLAG-cGAS and FLAG-STING were gifts from Dr. Daniel Lamarre (Université de Montréal).

HEK293T cells transfections were carried out using calcium-phosphate precipitation method unless otherwise stated. HeLa cells were transfected using Lipofectamine 2000 (Invitrogen) according to manufacturer’s protocol.

Sendai virus (SeV) was obtained from Specific Pathogen-Free Avian Supply (Charles River Laboratories). Cells were infected respecting the ratio of 100 HAU/10^6^ cells. GFP-expressing VSV (VSV-GFP, kindly provided by Dr. Benjamin tenOever, Mount Sinai Hospital, New York, NY, USA*)* was propagated in Vero cells and quantified by standard plaque assay as described here [120], and used at corresponding multiplicity of infection (MOI).

### Protein extraction and preparation of cytoplasmic and nuclear proteins

Proteins from whole cell extracts (WCE) were obtained by resuspending cells in conventional Triton X-100 lysis buffer complemented with protease inhibitors (50 mM Tris, pH 7.4; 150 mM NaCl; 50 mM NaF; 5 mM EDTA; 10% glycerol; 1 mM Na_3_VO_4_; 40 mM β-glycerophosphate; 0.1 mM phenylmethylsulfonyl fluoride; 5 μg/ml of leupeptin, pepstatin, and aprotinin; 1% Triton X-100) for 30 minutes on ice before being centrifuged and harvested. Proteins from WCE were quantified by Bradford protein assay (BioRad) according to manufacturer’s protocol.

For preparation of cytoplasmic and nuclear fractions, cells were harvested with ice-cold phosphate-buffered saline (PBS) and lysed by douncing 20 times in 500 μl membrane lysis buffer (10 mM, pH 7.9, Hepes, 10 mM KCl, 0.1 mM EDTA, 0.4% Nonidet P-40) containing protease inhibitors. The homogenate was centrifuged at 500 *g* for 10 min. The supernatant was saved as cytosol, and the pellet was saved as crude nuclei. The crude nuclei were washed twice with 500 μl membrane lysis buffer and resuspended in 20–50 μl of extract buffer (20 mM, pH 7.9, Hepes, 0.4 M NaCl, 1 mM EDTA) and shaken vigorously every 30 s for 15 min, followed by centrifugation at 15,000 *g* for 10 min. The supernatants containing nuclear proteins were saved for subsequent analysis.

### Immunoprecipitation, gel electrophoresis and immunoblot analysis

For co-immunoprecipitation assay, 1 mg of WCE were incubated with 1 μg of antibody at 4°C overnight on the rotating wheel, while 40 μl of protein A-Sepharose beads suspension were blocked with 1% BSA buffer. Lysates were then immunoprecipitated with corresponding sepharose beads at 4°C for 3 hours. After five washes with protease inhibitors complemented lysis buffer, immune complexes were recovered from beads with 50 μl 2X sample buffer before analysis by SDS-PAGE and immunoblotting.

Immunoblots were accomplished according to previously described procedures [121]. Succinctly, WCE (50 μg) were prepared in 1x sample buffer and separated using sodium dodecyl sulfate polyacrylamide gel electrophoresis (SDS-PAGE) method and a SE400 electrophoresis apparatus (GE Health Care). The proteins were transferred onto a nitrocellulose membrane (BioTrace NT) using Trans-Blot Electrophoretic Transfer Cell (Bio-Rad) according to manufacturer’s protocol. Nitrocellulose membranes were then incubated with 5% milk or 5% bovine serum albumin (BSA; for detection of phosphorylated proteins) for 1h to prevent non-specific binding of antibodies. For anti-p-mTOR-Ser2159, the membranes were blocked in 3% milk. The membranes were probed with primary antibodies followed by HRP-conjugated secondary antibodies raised against the appropriate species diluted in blocking buffer at a final concentration recommended by manufacturers. Bands were detected with the Western Lightning ECL kit (Perkin-Elmer). Densitometry analysis was performed using ImageJ 1.53 [122].

For Native-PAGE analysis, non-denaturing (without SDS) conditions were used. 7.5% native polyacrylamide gels were pre-run using only 25 mM Tris and 192 mM glycine buffer with corrected-pH of 8.4 in the anode chamber, but containing also 1% deoxycholate in the cathode chamber for 30 minutes at 40 mA. WCE (20 μg) were diluted in native sample buffer (62.5 mM Tris-HCl, pH 6.8, 15% glycerol, and bromophenol blue) and ran into native gel for 3 hours at 25 mA using the SE400 electrophoresis apparatus. Transfer and immunoblotting were accomplished as mentioned above.

### Confocal immunofluorescence microscopy

Confocal immunofluorescence microscopy experiments were accomplished as previously described by us [48]. Briefly, cells were fixed with 4% paraformaldehyde (PFA) in PBS for 20 min before being permeabilized through 5 minute-treatment with 0.1% Triton X-100. Cells were then washed with PBS (pH 7.2) and blocked with 0.5% BSA in PBS before being probed with primary antibodies and subsequent secondary fluorophore-conjugated antiserum (Alexa Fluor 488 and 564). Anti-FLAG antibody (M2, Sigma) was used at 1:1000, anti-FLAG polyclonal antibody; 1:400, anti-Myc 9E10; 1:100, anti-Sec31A; 1:100, anti-ERGIC-53; 1:100, anti-GM130; 1:100, anti-EEA1; 1:100, anti-Sec16A; 1:200, and anti-MAVS; 1:100. Secondary fluorophore-conjugated antiserum (Alexa Fluor 488 and 564) was used at 1:500 in PBS 0.5% BSA. The nucleus was labeled by 4′,6-diamidino-2-phenylindole (DAPI) staining. The confocal micrographs represent a single optical section (Z-stack) of cells. Images were acquired from a LSM 510 inverted microscope (Zeiss) combined to LSM v3.2 software (Zeiss). Colocalization of labeled protein was assessed by linescan analysis using “Profile” function in the ZEN 3.1 blue software (Zeiss). The pixel intensity in each channel is measured along a line drawn on the image and is plotted versus distance along the line.

### RNA interference

ON-TARGETplus siRNA against TFG mRNA (siTFG) and the non-targeting control (siNT) were purchased from Dharmacon. siRNA targeting the open reading frame are as follow: siTFG #1 (Cat# J-016366-08-0002), siTFG #2 (Cat# J-016366-07-0002), siTFG #3 (Cat# J-016366-06-0002), siTFG #4 (Cat# J-016366-05-0002). Cells were transfected with 40 nM siRNA using Lipofectamine 2000 at a final concentration of 4 μg/mL and maintained in culture for 72 hours before analyzing cell extracts. The RNAi Consortium (TRC)/ Mission shRNA lentiviral vectors targeting TFG (#1: TRCN0000078659; #2: TRCN0000078660; #3: TRCN0000311703) and non-targeting control (shNT: SHC002) shRNA were purchased from Sigma. Lentiviral vector production was conducted in HEK293T cells. Cells (3.5 x10^6^ in 100mm dish) were transfected with 6 μg of non-targeting control, specific shRNA along with 1.5 μg pMDLg/pRRE, 1.5 μg pRSV-REV, and 3 μg pVSVg using Lipofectamine 2000 (Invitrogen). 16 hours post-transfection, the medium was replaced before being harvested the next day. Medium containing lentivirus was then filtered through 0.45 μm filter and stored at -80°C. Lentiviral titers were determined by limiting dilution assay using HeLa cells as described [123]. The cells were infected with lentivirus at an MOI of 5 for 24h in the presence of 8 μg/ml polybrene followed by puromycin selection for 3 days (2 μg/ml) before further manipulation.

### *In vitro* kinase assay

The phosphotransferase activity of the TBK1 was assayed as described previously [124]. Whole-cell extracts (1000 μg) were incubated with 60 μL protein G-Sepharose beads pre-adsorbed with FLAG M2 beads overnight at 4°C. Beads were washed 3 times with ice-cold lysis buffer and 1 time with kinase assay buffer (20 mM HEPES, pH 7.4, 20 mM MgCl2, 2 mM dithiothreitol, and 20 μM NaO4). Beads were resuspended in kinase assay buffer containing 100 or 500 ng of recombinant full length human TBK1 (Upstate Biotechnology, Lake Placid, NY, Cat# 14–628), 20μM of ATP and 20μCi of [γ-^32^P]ATP. The kinase reactions were incubated at 30°C for 30 min and stopped by the addition of 5X Laemmli's sample buffer and heating at 95°C for 10 min. The reactions were resolved on 6% SDS-PAGE and the gels were dried and exposed for autoradiography for imaging with Typhoon scanner 9410 (Amersham Biosciences) or transferred onto a nitrocellulose membrane for immunoblot analysis.

### RNA isolation and RT-qPCR analysis

Total RNA from MRC5 was extracted by using the RNeasy mini Kit (Qiagen). RNA was quantified with NanoPhotometer (Implen GmbH, Munich, Germany), and samples were evaluated for integrity with a 2100 Bioanalyzer (Agilent Technologies, Palo Alto, CA). RNA was reverse transcribed into cDNA with the Maxima First Strand cDNA synthesis kit with dsDNase (Thermo Fisher Scientific). Gene expression was determined using assays designed with the Universal Probe Library from Roche (www.universalprobelibrary.com). For each qPCR assay, a standard curve was performed to ensure that the efficiency of the assay was between 90% and 110%. The QuantStudio7 qPCR instrument (Thermo Fisher Scientific) was used to detect the amplification level. All reactions were run in triplicate and Relative mRNA expression was calculated according to the comparative threshold (*C*_*T*_) formula 2^−ΔΔ*CT*^, where ΔΔ*C*_*T*_ = Δ*C*_*T*_ test sample–Δ*C*_*T*_ calibrator sample and Δ*C*_*T*_ = *C*_*T*_(target)–*C*_*T*_(endogenous control). *HPRT* and *TBP* were used as endogenous control. The sequences of the primers and Universal Probe Library (UPL) probes used are listed in S1 Table.

### ELISA

IFN-β production and secretion in supernatants was determined using the Verikine human IFN beta ELISA kit (Cat# 41410, PBL Assay Science) according to manufacturer's instructions.

### VSV-GFP antiviral assay and flow cytometry analysis

The antiviral state of cells following TFG knockdown was measured by VSV-GFP reporter virus replication as described previously [125]. Briefly, cells were infected with VSV-GFP and cells were inspected and photographed using an inverted fluorescence microscope (Zeiss, Goettingen, Germany) 16h post-infection. Fluorescence intensity was quantified using ImageJ 1.53 [122]. Moreover, VSV-GFP infected cells were trypsinized and either harvested for immunoblot analysis or fixed with 2% paraformaldehyde in PBS and analyzed by flow cytometry using FACS caliber (BD Bioscience) combined with the BD FACSDiva software.

### Statistical analysis

Statistical analyses were performed using GraphPad Prism version 5.0. All data are from a minimum of two independent experiments. Comparison of two groups was carried out using a two-tailed t-test, and comparison of more than two groups was evaluated with one-way ANOVA and Bonferroni test for multiple comparisons. Differences were considered significant at a P-value below 0.05.

## Supporting information

S1 Fig (A) Representative linescan analysis of confocal data showing TFG-TRAF3 colocalization presented in Fig 1D. The pixel intensity in each channel is measured along a line drawn on the image and is plotted versus distance along the line. (B-C) HeLa cells were transfected with both Myc-TFG and FLAG-TRAF3. Cells were stained with anti-Myc (9E10) and polyclonal anti-FLAG antibodies. Nuclei were labeled with DAPI. Cells were then visualized by confocal microscopy. Images are representative of three independent experiments in which cells were examined and displayed similar staining. Data for 2 cells are shown with a representative linescan analysis shown below.
(TIF)

S2 Fig Representative linescan analysis of confocal data showing colocalization of TFG and different markers of perinuclear compartments presented in Fig 2. The pixel intensity in each channel is measured along a line drawn on the image and is plotted versus distance along the line.
(TIF)

S3 Fig (A) Densitometry analysis of immunoprecipitated TRAF3 interacting with TFG. Data from two independent experiments presented in Fig 3A were quantified and input-normalized TRAF3 signal is shown. (B) Densitometry analysis of IRF3 dimerization following SeV infection of siNT and siTFG cells. Data from two independent experiments presented in Fig 5A were quantified and α-tubulin- normalized dimer signal is shown. (C) Densitometry analysis of pIRF3 following SeV infection of shNT and shTFG cells. Data from three independent experiments presented in Fig 5B were quantified and the β-actin-normalized signal is shown.
(TIF)

S4 Fig Representative linescan analysis of confocal data showing colocalization of TRAF3 and MAVS presented in Fig 3C. The pixel intensity in each channel is measured along a line drawn on the image and is plotted versus distance along the line. NS (A), SeV (B), and Poly:(IC) (C)
(TIF)

S5 Fig No significant effect of TFG knockdown on the phosphorylation of TBK1 in response to DNA sensor agonists. THP-1 monocytes were transfected with an siRNA duplex (NT or TFG) and three days post-transfection, cells stimulated with the DNA sensor agonists Poly dA:dT (2 μg/ml), ISD (2 μg/ml), cGAMP (5 μg/ml) and VACV-70 (2 μg/ml) for indicated time. Whole cell extracts (WCE) were harvested and subjected to immunoblot analysis with indicated antibodies.
(TIF)

S6 Fig Effect of an mTOR inhibitor on SeV-induced ISGs expression. Serum starved primary MRC5 fibroblasts were pretreated with 0.5 μM Ku-0063794, a highly selective mTOR inhibitor, or vehicle for 30 minutes and then left uninfected or infected with SeV (100 HAU/10^6^ cells) for the indicated times in the continuous presence of the drug. RNA was extracted and analyzed by RT-qPCR for indicated gene expression. Mean values and SD of two independent experiments are shown. RQ, relative quantification.
(TIF)

S7 Fig Activation of mTORC1 and the phosphorylation of mTOR on Ser2159 do not prevent the nuclear accumulation of IRF3. A) MRC5 were infected with SEV (100 HAU/10^6^ cells) for 6 hours under the continuous presence of DMSO or Rapamycin [20 ng/ml]. B) 293T cells were transfected with the indicated constructs. 24h post-transfection, crude nuclear and cytoplasmic fractions were prepared to perform immunoblot analysis with the indicated antibodies.
(TIF)

S1 Table RT-qPCR probes and primers used in this study. (DOCX)
