# Supplementary figures and images for: TRK-Fused Gene (TFG), a protein involved in protein secretion pathways, is an essential component of the antiviral innate immune response
Source: PLoS Pathog. 2021 Jan 7;17(1):e1009111. doi: 10.1371/journal.ppat.1009111 (PMC7790228; doi:10.1371/journal.ppat.1009111)

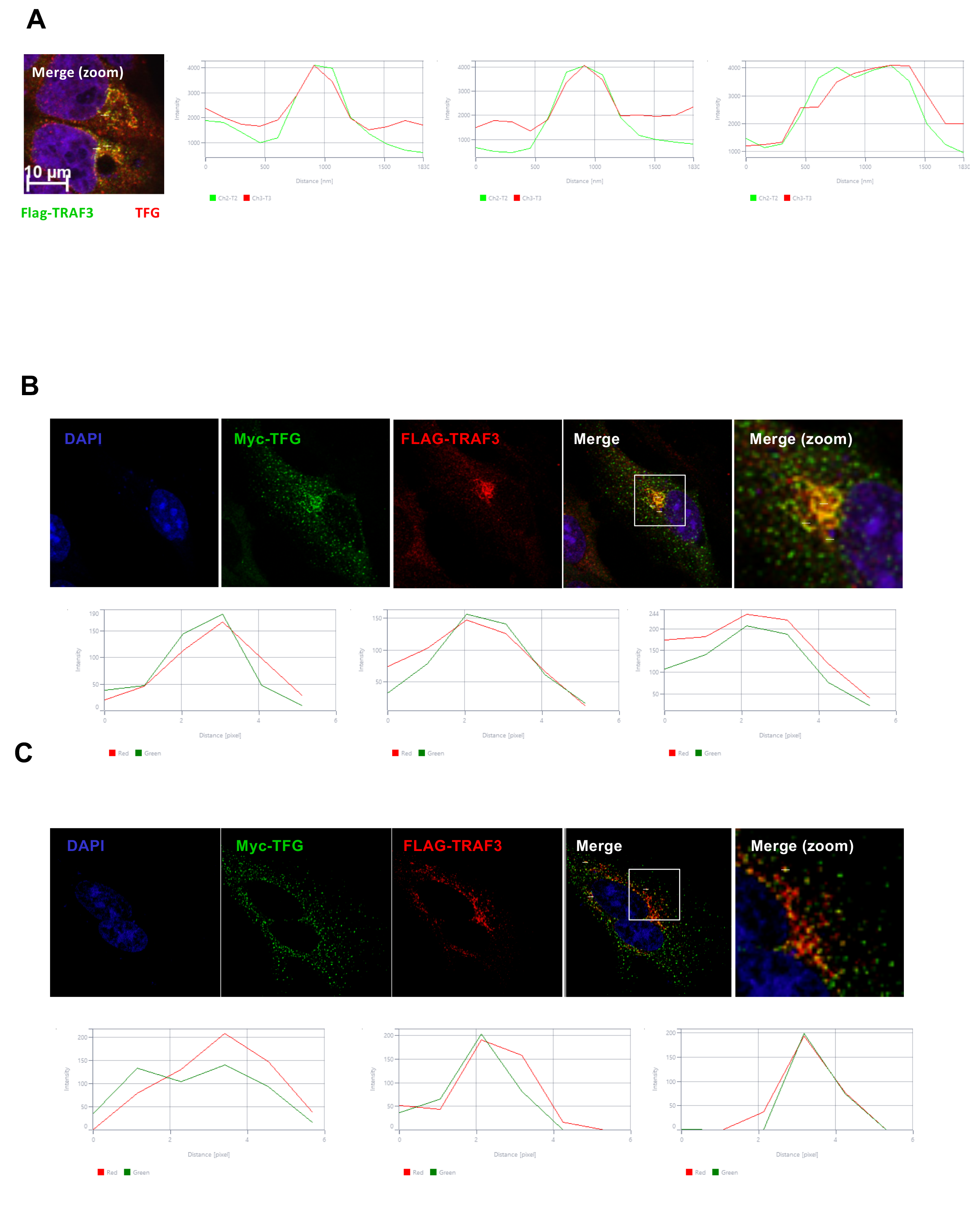

Supplement: S1 Fig — (A) Representative linescan analysis of confocal data showing TFG-TRAF3 colocalization presented in Fig 1D. The pixel intensity in each channel is measured along a line drawn on the image and is plotted versus distance along the line. (B-C) HeLa cells were transfected with both Myc-TFG and FLAG-TRAF3. Cells were stained with anti-Myc (9E10) and polyclonal anti-FLAG antibodies. Nuclei were labeled with DAPI. Cells were then visualized by confocal microscopy. Images are representative of three independent experiments in which cells were examined and displayed similar staining. Data for 2 cells are shown with a representative linescan analysis shown below. (TIF) [file ppat.1009111.s001.tif]

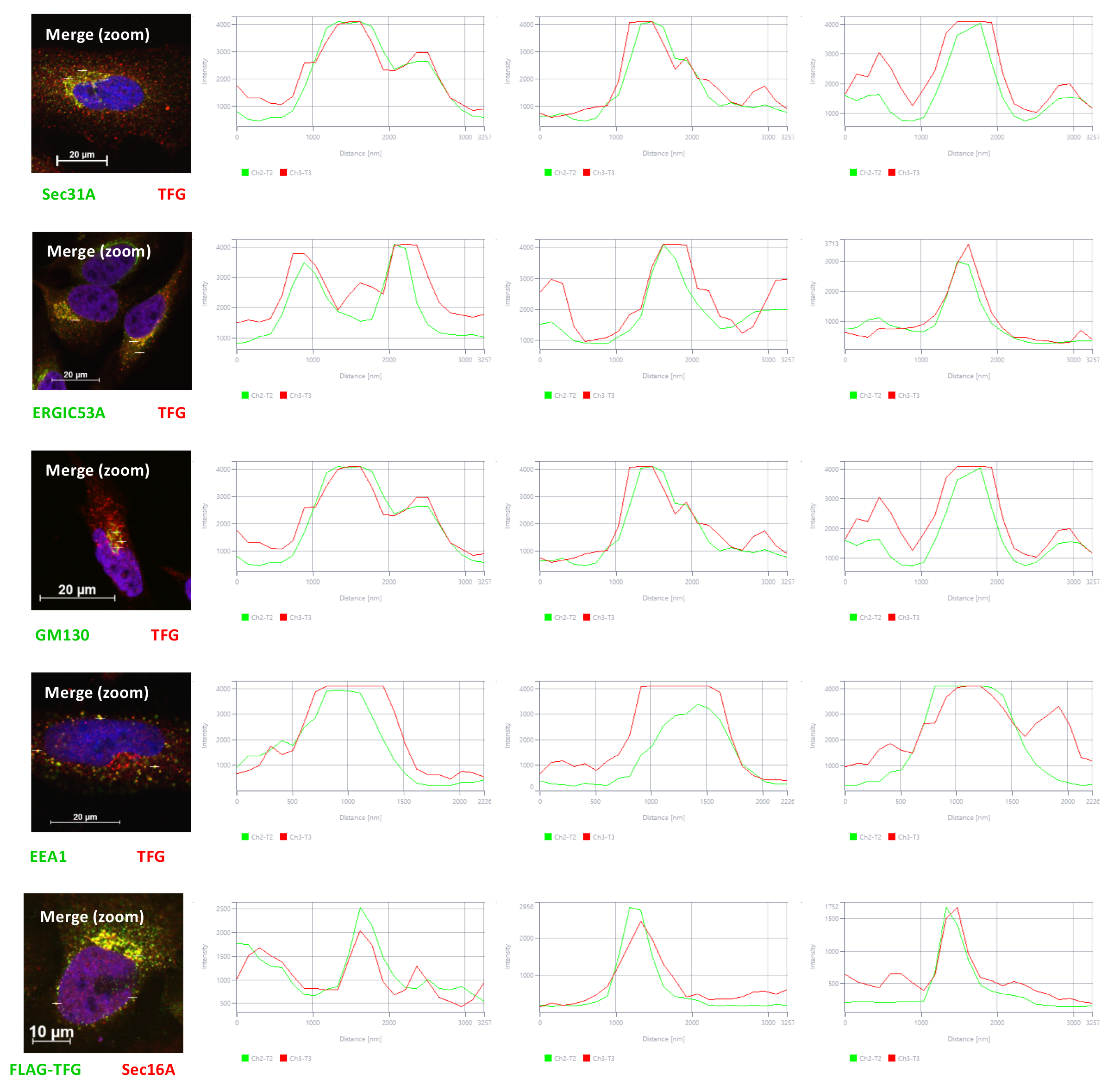

Supplement: S2 Fig — The pixel intensity in each channel is measured along a line drawn on the image and is plotted versus distance along the line. (TIF) [file ppat.1009111.s002.tif]

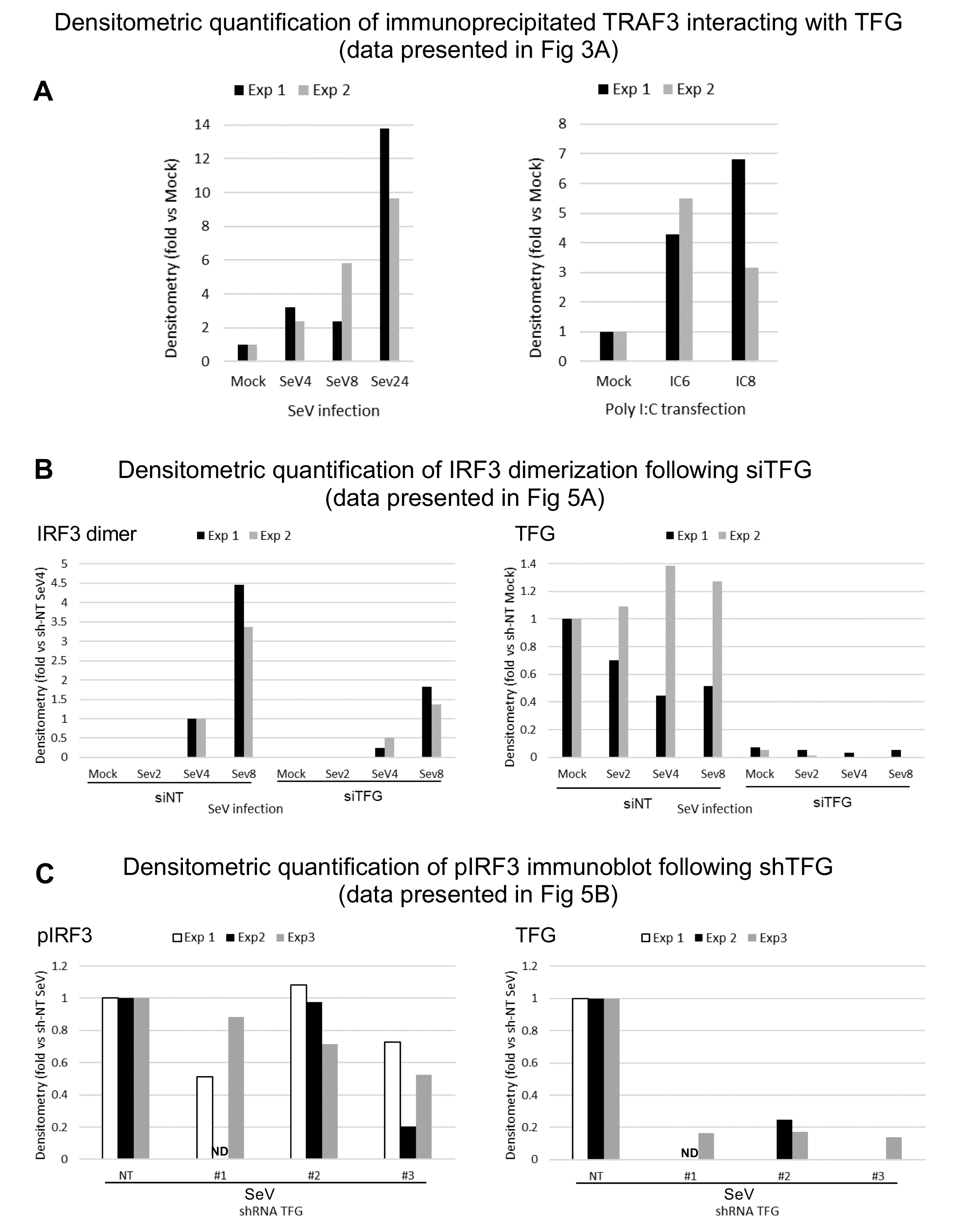

Supplement: S3 Fig — (A) Densitometry analysis of immunoprecipitated TRAF3 interacting with TFG. Data from two independent experiments presented in Fig 3A were quantified and input-normalized TRAF3 signal is shown. (B) Densitometry analysis of IRF3 dimerization following SeV infection of siNT and siTFG cells. Data from two independent experiments presented in Fig 5A were quantified and α-tubulin- normalized dimer signal is shown. (C) Densitometry analysis of pIRF3 following SeV infection of shNT and shTFG cells. Data from three independent experiments presented in Fig 5B were quantified and the β-actin-normalized signal is shown. (TIF) [file ppat.1009111.s003.tif]

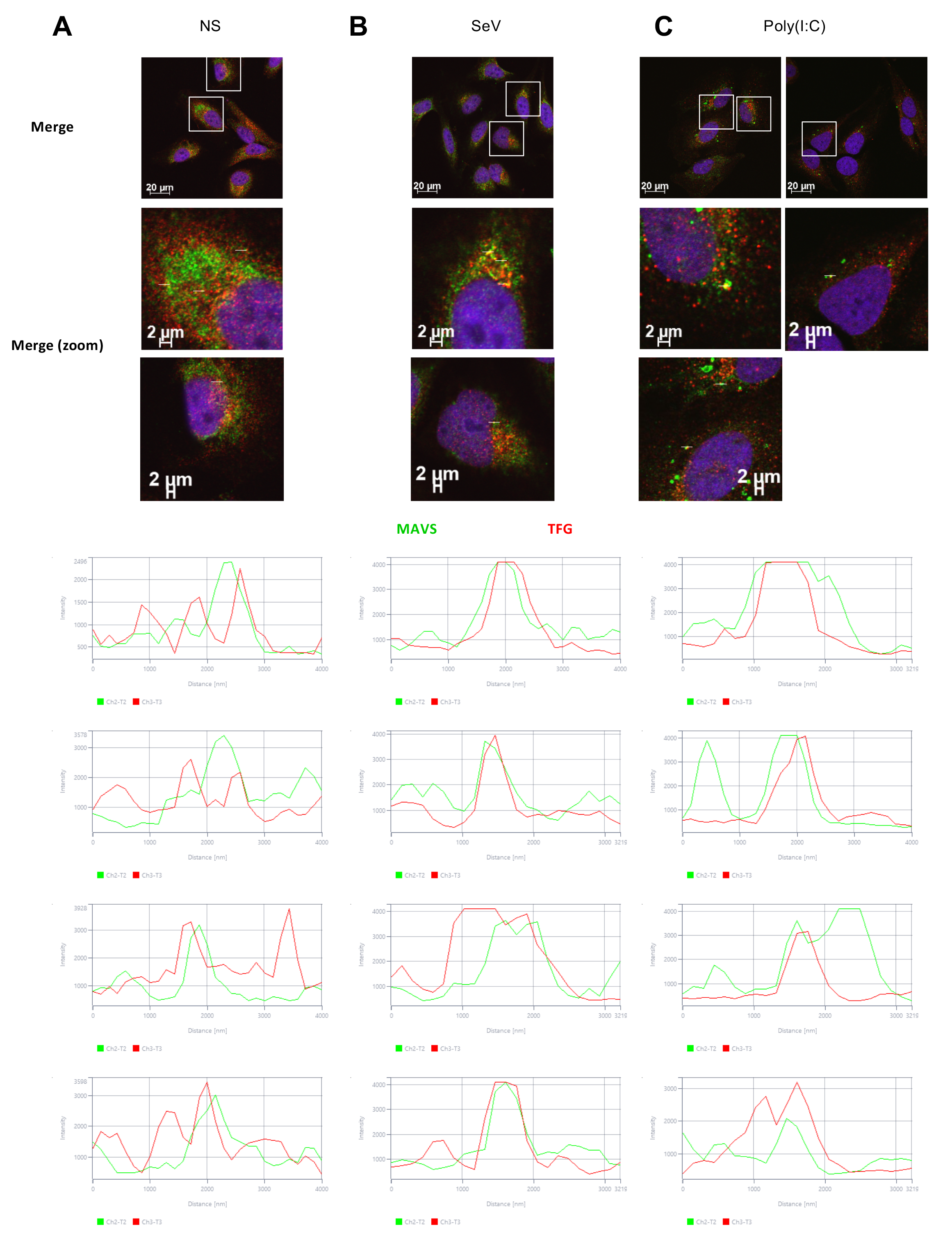

Supplement: S4 Fig — The pixel intensity in each channel is measured along a line drawn on the image and is plotted versus distance along the line. NS (A), SeV (B), and Poly:(IC) (C) (TIF) [file ppat.1009111.s004.tif]

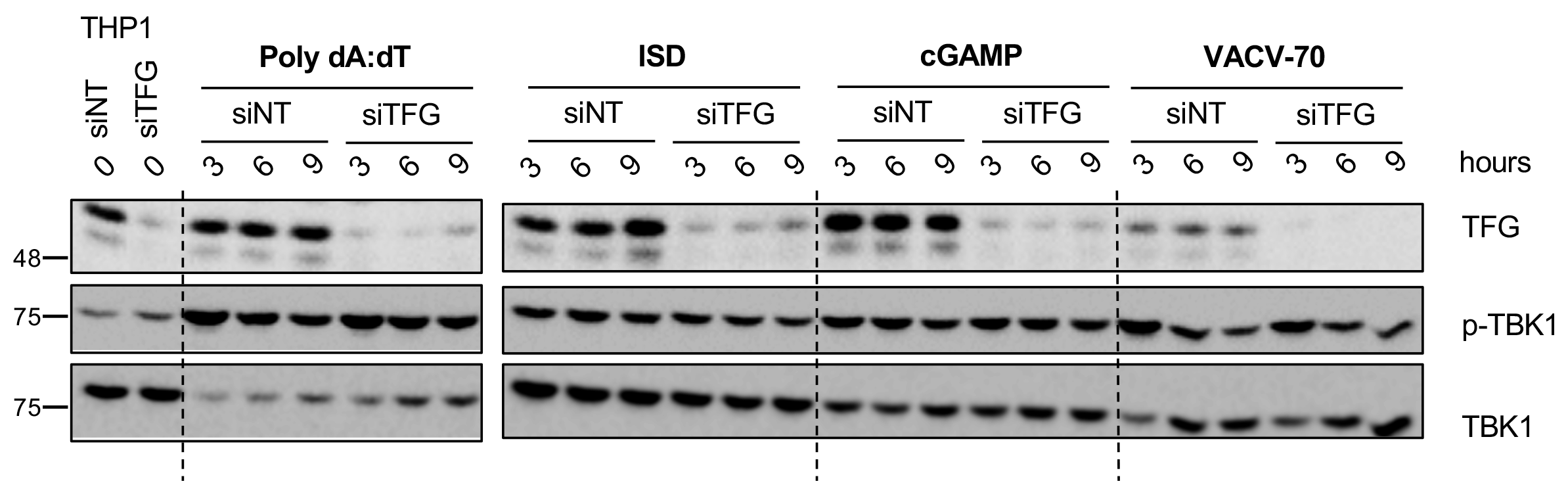

Supplement: S5 Fig — THP-1 monocytes were transfected with an siRNA duplex (NT or TFG) and three days post-transfection, cells stimulated with the DNA sensor agonists Poly dA:dT (2 μg/ml), ISD (2 μg/ml), cGAMP (5 μg/ml) and VACV-70 (2 μg/ml) for indicated time. Whole cell extracts (WCE) were harvested and subjected to immunoblot analysis with indicated antibodies. (TIF) [file ppat.1009111.s005.tif]

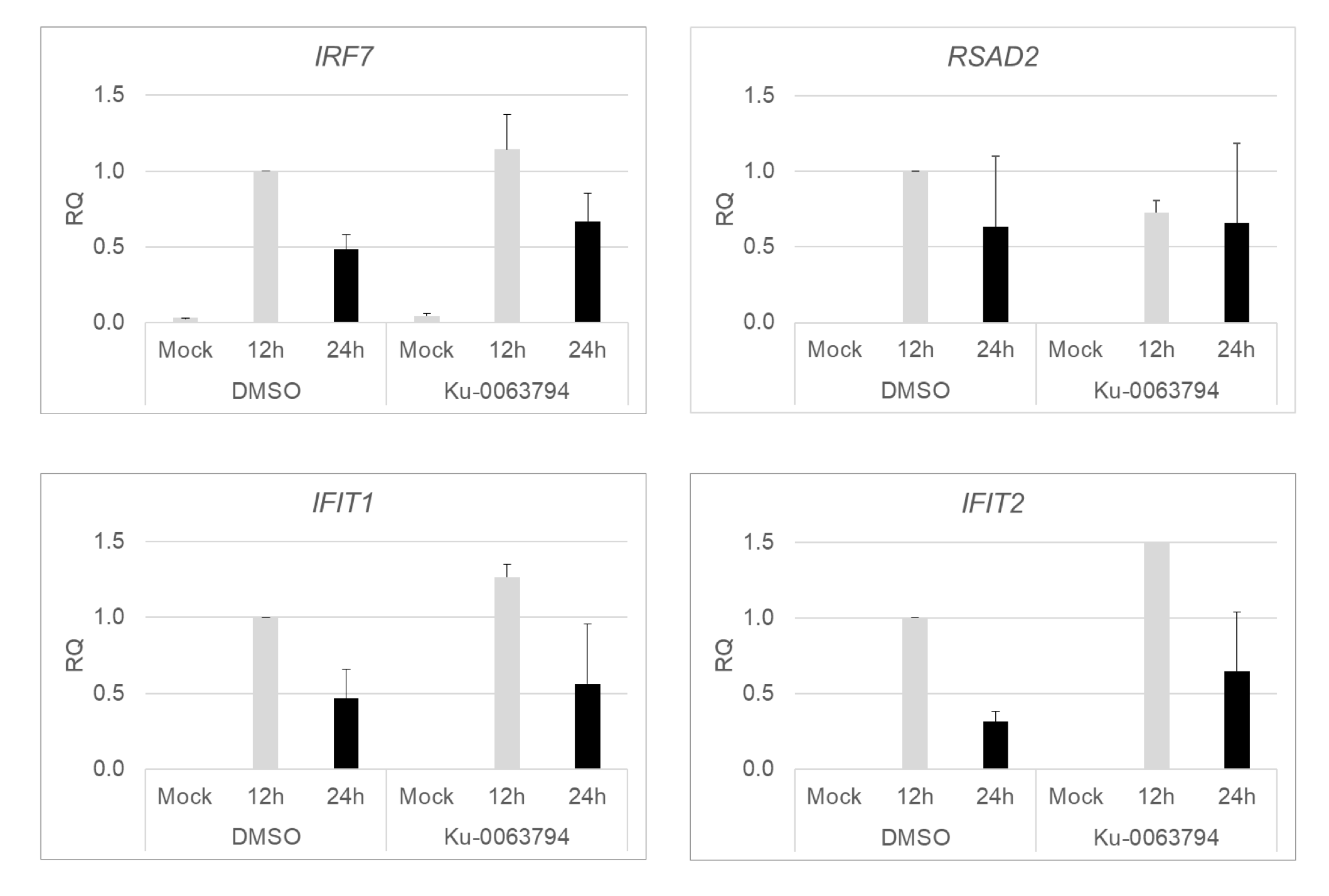

Supplement: S6 Fig — Serum starved primary MRC5 fibroblasts were pretreated with 0.5 μM Ku-0063794, a highly selective mTOR inhibitor, or vehicle for 30 minutes and then left uninfected or infected with SeV (100 HAU/106 cells) for the indicated times in the continuous presence of the drug. RNA was extracted and analyzed by RT-qPCR for indicated gene expression. Mean values and SD of two independent experiments are shown. RQ, relative quantification. (TIF) [file ppat.1009111.s006.tif]

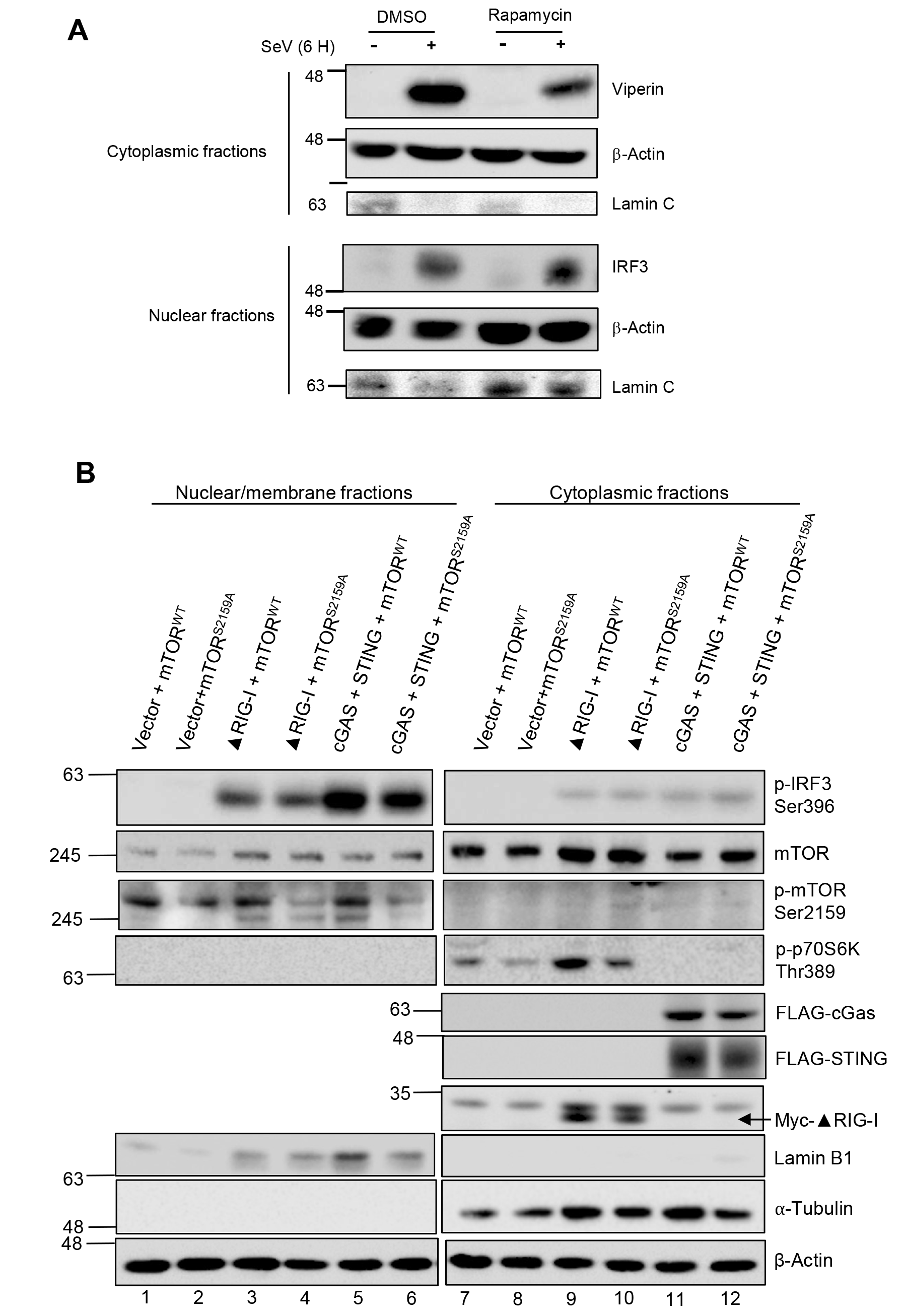

Supplement: S7 Fig — A) MRC5 were infected with SEV (100 HAU/106 cells) for 6 hours under the continuous presence of DMSO or Rapamycin [20 ng/ml]. B) 293T cells were transfected with the indicated constructs. 24h post-transfection, crude nuclear and cytoplasmic fractions were prepared to perform immunoblot analysis with the indicated antibodies. (TIF) [file ppat.1009111.s007.tif]
