# Supplementary material for: TRK-Fused Gene (TFG), a protein involved in protein secretion pathways, is an essential component of the antiviral innate immune response
Source: PLoS Pathog. 2021 Jan 7;17(1):e1009111. doi: 10.1371/journal.ppat.1009111 (PMC7790228; doi:10.1371/journal.ppat.1009111)
Supplement: S1 Table — (DOCX) [file ppat.1009111.s008.docx]

**S1 Table: RT-qPCR probes and primers used in this study**

| **Gene** | **Ref Seq accession no.** | **UPL Probe** | **Primer sequence** | |
| --- | --- | --- | --- | --- |
|  |  |  | **Forward** | **Reverse** |
| *IFNB1* | NM_002176.2 | 25 | cgacactgttcgtgttgtca | gaagcacaacaggagagcaa |
| *ISG15* | NM_005101.3 | 76 | gcgaactcatctttgccagt | ttcagctctgacaccgacat |
| *IFIT1* | NM_001548.3 | 9 | agaacggctgcctaatttacag | gctccagactatccttgacctg |
| *RSAD2* | NM_080657.4 | 39 | tgcttttgcttaaggaagctg | aggtattctccccggtcttg |
| *IFIT2* | NM_001547.4 | 27 | tggtggcagaagaggaagat | gtaggctgctctccaaggaa |
| *CCL5* | NM_002985.2 | 59 | tgcccacatcaaggagtattt | gggtgacaaagacgactgct |
| *CXCL10* | NM_001565.4 | 34 | gaaagcagttagcaaggaaaggt | gacatatactccatgtagggaagtga |
| *IFNL1* | NM_172140.1 | 65 | gggacctgaggcttctcc | ccaggaccttcagcgtca |
| *IFNL2* | NM_172138.1 | 22 | cctggtggacgtcttgga | gtgggctgaggctggata |
| *IFNL3* | NM_172139.2 | 60 | agggccaaagatgccttag | cagctcagcctccaaagc |
| *IRF7* | NM_004031.4 | 76 | agagcctggtcctggtgaa | ctgcgtgccctctaggtg |
| *HPRT* | NM_000194.2 | 22 | tgatagatccattcctatgactgtaga | caagacattctttccagttaaagttg |
| *TBP* | NM_003194.3 | 87 | gaacatcatggatcagaacaaca | atagggattccgggagtcat |
